# Soil Microbial Responses to Varying Environmental Conditions in a Copper Belt Region of Africa: Phytoremediation Perspectives

**DOI:** 10.3390/microorganisms13010031

**Published:** 2024-12-27

**Authors:** Kabwe Nkongolo, John B. Mukalay, Antoine K. Lubobo, Paul Michael

**Affiliations:** 1 School of Natural Sciences, Laurentian University, 935 Ramsey Lake Road, Sudbury, ON P3E 2C6, Canada; pmichael@laurentian.ca; 2 Faculty of Agronomy, University of Lubumbashi, Lubumbashi BP 1825, Democratic Republic of the Congo; mukalayjohn@gmail.com (J.B.M.); lubobo@unilu.ac.cd (A.K.L.); 3 Water, Soil and Plant Exchanges, Gembloux Agro-Bio Tech, University of Liège, 5030 Gembloux, Belgium

**Keywords:** copper belt region, Lubumbashi, Illumina sequencing, metal translocation from rhizosphere, microbial abundance and diversity, phytoremediation

## Abstract

The mining industry in the copper belt region of Africa was initiated in the early 1900s, with copper being the main ore extracted to date. The main objectives of the present study are (1) to characterize the microbial structure, abundance, and diversity in different ecological conditions in the cupriferous city of Lubumbashi and (2) to assess the metal phytoextraction potential of *Leucaena leucocephala*, a main plant species used in tailing. Four ecologically different sites were selected. They include a residential area (site 1), an agricultural dry field (site 2), and an agricultural wetland (site 3), all located within the vicinity of a copper/cobalt mining plant. A remediated tailing was also added as a highly stressed area (site 4). As expected, the highest levels of copper and cobalt among the sites studied were found at the remediated tailing, with 9447 mg/kg and 2228 mg/kg for copper and cobalt, respectively. The levels of these metals at the other sites were low, varying from 41 mg/kg to 579 mg/kg for copper and from 4 mg/kg to 110 mg/kg for cobalt. Interestingly, this study revealed that the *Leucaena leucocephala* grown on the remediated sites is a copper/cobalt excluder species as it accumulates soil bioavailable metals from the rhizosphere in its roots. Amplicon sequence analysis showed significant differences among the sites in bacterial and fungal composition and abundance. Site-specific genera were identified. *Acidibacter* was the most abundant bacterial genus in the residential and remediated tailing sites, with 11.1% and 4.4%, respectively. *Bacillus* was predominant in both dry (19.3%) and wet agricultural lands (4.8%). For fungi, *Fusarium* exhibited the highest proportion of the fungal genera at all the sites, with a relative abundance ranging from 15.6% to 20.3%. Shannon diversity entropy indices were high and similar, ranging from 8.3 to 9 for bacteria and 7.0 and 7.4 for fungi. Β diversity analysis confirmed the closeness of the four sites regardless of the environmental conditions. This lack of differences in the microbial community diversity and structures among the sites suggests microbial resilience and physiological adaptations.

## 1. Introduction

Land use change affects the biodiversity and function of ecosystems [1,2,3]. Microorganisms play a crucial role in ecosystem functioning, and changes in land use can significantly affect microbial diversity. Forests support a wide range of microbial communities including bacteria, fungi, and archaea, which contribute to decomposition, nutrient cycling, and carbon sequestration [4]. Likewise, grasslands host diverse microbial communities adapted to varied soil types, influencing soil fertility and plant productivity [5]. Wetlands, on the other hand, harbor unique microbial communities involved in nutrient cycling, pollution mitigation, and carbon storage [6]. Mining activities increase the levels of metals in soils, which affect microbial communities’ structure and function [7]. In general, sources of metal contamination include industrial activities such as mining and smelting; agriculture practices, including fertilizers and pesticides; urbanization (vehicle emissions, construction); and waste disposal [8]. Metals affecting microbial communities include heavy metals (Pb, Cd, Hg, As), transition metals (Cu, Zn, Ni), and metalloids. Soil pH, total and available metals, and organic matter (SOM) impact soil enzyme activities and microbial composition [7].

In most mining regions, tailings are developed from mining operations or industrial waste [9,10]. These sites are highly contaminated with toxic metals that generate stress on organisms and limit the development of healthy biological processes [9,11,12]. Studies on microbial changes induced by accumulations of metals such as in tailings or industrial waste areas are limited.

At the ecological level, several studies have investigated how the conversion of forest to agricultural sites affects soil properties [13,14]. However, reports on the effects on ecological function of forest soil lands’ conversion to industrial fields, especially in cases of metal contamination, are limited. The process defining how soil metal contamination change drives the microbial community structure is still poorly understood. It is known, however, that urbanization decreases microbial richness and evenness and favors tolerant and opportunistic microorganisms. Microbial populations can adapt to urban environmental pressures [15].

High levels of metals from mining operations represent an important environmental concern in the copper belt region of Africa since metallic pollutants are significantly toxic, even in very small amounts [16,17]. To reduce damage in metal-impacted lands, phytoremediation programs have been initiated in some tailings in the CBRA. Several plant species have been used for restoration of metal-contaminated tailings and landfill sites in the Copper Belt region. Their coping mechanisms for soil metal toxicity has not been Investigated in detail. *Leucaena leucocephala* is one of the main species used for the phytoremediation of metal-contaminated lands in mining areas in Lubumbashi. Its phytoremediation potential has not been demonstrated. This species built symbiotic associations with rhizobia in a mine tailing contaminated with iron-vanadium-titanium oxide. This symbiosis resulted in reduced metal uptake for *Leucaena leucocephala* grown in mine tailings and metal-polluted soils in China [18]. Discussions of the impact of environmental stressors on soil microbial communities in different studies conducted worldwide remain unsettled. In general, metal ions affect soil microbial activities by interfering with important processes [19]. Understanding the underlying differences in soil bacterial diversity and community structures under different environmental conditions is of great interest [20].

The specific objectives of this study are to (1) characterize soil bacterial and fungal structure, abundance, and diversity in different ecological conditions in the copper belt city of Lubumbashi and (2) to assess the potential of *Leucaena leucocephala*, a main plant species used in tailings in Lubumbashi, to extract metals from the soil rhizosphere to its roots and other plant tissues.

## 2. Materials and Methods

### 2.1. Sampling and Site Characterization

**Figure 1:**
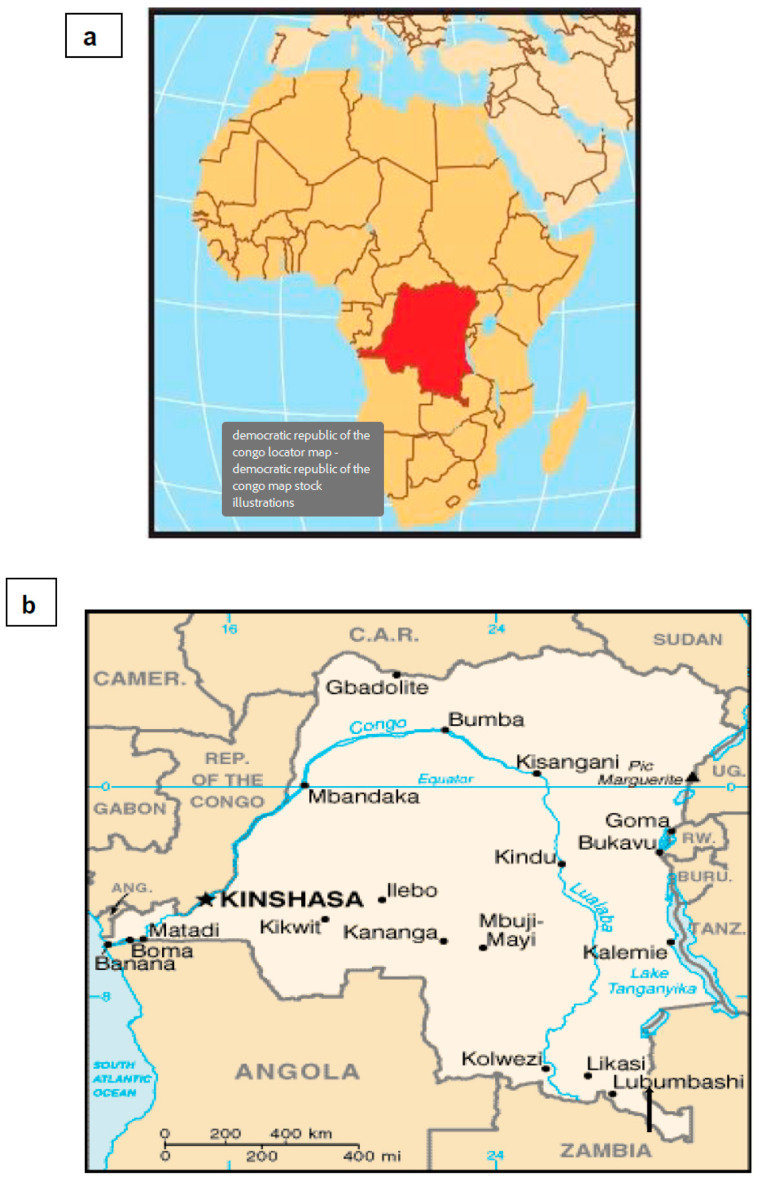
Location of soil sampling sites. (**a**) Democratic Republic of Congo (red) on a map of Africa; (**b**) details in the map of Democratic Republic of Congo. The arrow indicates the sites’ location (Lubumbashi). Adapted from Google Map, accessed in June 30, 2023. Site 1—Urban area (11.644767 S, 27.557596 E), Site 2—agricultural dry land (11.66034 S, 27.556109 E); Site 3—agricultural wetland (11.656886 S, 27.575442 E), Site 4—remediated tailing (11.608833 S, 27.476675 E).

Soil samples were collected from four sites (Figure 1). Four ecologically different sites located in the copper city of Lubumbashi were selected. They include a residential area (site 1), an agricultural dry field (site 2), and an agricultural wetland (site 3) located within the vicinity of a copper/cobalt mining plant. A remediated tailing was also added as a highly stressed area (site 4). The agricultural field (site 2) is used to produce corn (*Zea mays*), sweet potatoes (*Ipomoea batata*), okra (*Abelmoschus esculentus*), beans (*Phaseolus vulgaris*), and peanuts (*Arachis hypogaea*). The site 3 wetland (market gardening) is used for amaranth (*Amaranthus* spp.), okra (*Abelmoschus esculentus* L.), head cabbages (*Brassica oleracea*), tomatoes (*Solanum lycopersicum*), onions (*Allium cepa*), eggplants (*Solanum melongena*), and cucumber (*Cucumis sativus*) cultures. The remediated site (site 4) was used to dump residues from industrial plants and household debris. This site covers an area of 2 ha (10,000 m^2^). *Leucaena leucocephala* has been grown for phytoremediation of this area.

The sampling design is as described in Nkongolo et al. (2022) [21]. At each site, soil samples were collected at three areas, each representing a replication. For each replication, a composite of 20 subsamples from the top layer (0–15 cm) was collected, mixed, and homogenized. Plant materials, stones, and other residues were removed using a KimLab Economy Test Sieve with a 2 mm mesh size (#10) (https://www.amazon.ca/KimLab-Economy-Stainless-Plating-Diameter/dp/B07KXYNC13?th=1 accessed on 22 December 2024).

### 2.2. Soil pH, Organic Matter, and Metal Analysis

The analysis for soil pH, organic matter (OM), and metal levels were performed at Testmark Inc. (Sudbury) as described in Nkongolo et al. [22,23]. To assess total metal concentrations, a 0.5 g soil sample was treated with 10 mL of 10:1 ratio HF:HCl, heated to 110 °C for 3.5 h in open 50 mL Teflon™ tube in a programmable digestion block to dry samples, followed by addition of 7.5 mL of HCl and 7.5 mL of HNO_3_ and heating to 110 °C for another 4 h to dry gently. The samples were then heated to 110 °C for 1 h following addition of 0.5 mL of HF, 2 mL of HCl, and 10 mL of HNO_3_ to reduce sample volume to 8–10 mL. On cooling, the samples were diluted to 50 mL with ultrapure water for subsequent analysis by plasma spectrometry. The bioavailable metals were also estimated according to Nkongolo et al. [22,23] by extracting 5 g of soil with 20 mL of 0.01 M LiNO_3_ in a 50 mL centrifuge tube in a shaker under ambient lighting conditions for 24 h at 20 °C [22,24]. The pH (LiNO_3_) of the suspension was measured prior to centrifugation at 3000 rpm for 20 min, with filtration of the supernatant through a 0.45 µm filter into a 20 mL polyethylene tube and diluted to volume with deionized water. The filtrate was preserved at approximately 3 °C for analysis by ICP-MS. The quality control process was as described in Nkongolo et al. [22,23].

### 2.3. Phytoremediation Potential of Leucaena leucocephala

The bioaccumulation factor for the main metals was determined as the ratio of total copper and cobalt amounts in roots over the bioavailable Cu and Co in the rhizosphere. Further, the translocation factor was calculated as the ratio of Cu and Co amount in *L. leucocephala* areal part tissues over root metal content.

### 2.4. Amplicon Sequencing and MiSeq

For metagenomics analysis, four areas with varying environmental conditions (levels of Cu and Co, pH, organic matter content, and land use) and extractable eDNA were selected. Microbial DNA was extracted from soil samples following the procedure described by Nkongolo et al. [9]. “This was followed by purification of genomic DNA, fragmentation, ligation to sequencing adapters, and purification. We used 50 ng of DNA from each sample to prepare the libraries using Nextera DNA Sample Preparation Kit (Illumina, San Diego, CA, USA). Library insert size was determined by Experion Automated Electrophoresis Station (Bio-Rad, Hercules, CA, USA). The insert size of the libraries ranged from 300 to 850 bp (average 500 bp). Pooled library (12 pM) was loaded in a 600 Cycles v3 Reagent cartridge (Illumina) and the sequencing was performed on MiSeq (Illumina)”.

“Amplicon sequencing was performed at MR DNA Molecular Research DNA laboratory (Shallowater, TX, USA). Amplicon-based analysis of the soil bacterial and fungal microbiota was assessed by high-throughput sequencing of 16S rRNA gene and internal transcribed spacer (ITS) region. The 16S rRNA gene V4 variable region PCR primers 515F (5′-GTGCCAGCMGCCGCGGTAA-3′) and 806R (5′-GGACTACHVGGGTWTCTAAT-3′) with barcode on the forward primer were used. For fungi, ITS-specific primers ITS1F-Bt1 (5′-CTTGGTCATTTAGAGGAAGTAA-3′) and ITS2R (5′-GCTGCGTTCTTCATCGATGC-3′) were used to amplify 600 bp fragments of the fungal ITS region [25]. The Illumina MiSeq with methods via the bTEFAP^®^ DNA analysis service were used. Each sample underwent a single-step 35 cycle PCR using HotStarTaq Plus Master Mix Kit (Qiagen, Valencia, CA, USA) under the following conditions: 95 °C for 5 min, followed by 30 cycles of 95 °C for 30 s; 53 °C for 40 s, and 72 °C for 1 min; after which a final elongation step at 72 °C for 10 min was performed. Following PCR, all amplicon products from different samples were mixed in equal concentrations and purified using calibrated SPRI beads. Samples were sequenced utilizing the Illumina MiSeq chemistry following manufacturer’s protocols”.

“The Q25 sequence data derived from the sequencing process were processed using the MR DNA ribosomal and functional gene analysis pipeline (MR DNA, Shallowater, TX, USA). Sequences were depleted of primers, short sequences < 150 bp were removed, and sequences with ambiguous base calls removed. Sequences were quality filtered using a maximum expected error threshold of 1.0 and dereplicated. The dereplicated or unique sequences were denoised; unique sequences identified with sequencing or PCR point errors were removed, followed by chimera removal, thereby providing a denoised sequence or zOTU. Final zOTUs were taxonomically classified using BLASTn against a curated database derived from NCBI and compiled in each taxonomic level into both “counts” and “percentage” files. Counts files contained the actual number of sequences, while the percent files contained the relative (proportion) percentage of sequences within each sample that map to the designated taxonomic classification”.

For statistical analysis, XLstat version 2021.5, NCSS 2007, “R (R-3.2.2)”, and NCSS 2010 packages were used to analyze alpha and beta diversities. These diversity measures were performed using QIIME 2 according to previous reports [26,27,28,29,30,31]. ANOVA and post hoc pairwise comparisons of the relative abundance of specific genera identified by the MRDNA pipeline were conducted using Tukey’s test. This was to determine if any specific genera were significantly different between treatment groups.

**Alpha diversity** data for observed features (amplicon sequence variants, ASVs) and Shannon diversity entropy indices (SDEIs) for each treatment group were conducted. SDEI provides a measure of the relative abundance of each ASV within a given site, capturing both evenness and abundance [32].

$$H=\sum_{i=1}^{s}-(P_{i}\times lnP_{i})$$

where

**H** = Shannon entropy

***Pi*** = fraction of the entire population made up of ASV i

ln***Pi*** = the natural log of above

***S*** = number of ASVs encountered

∑ = sum from ASV 1 to ASV S

Pielou’s measure of species evenness was estimated based on J = H′/ln(S). H′ is Shannon–Weiner diversity, and S is the total number of features in a sample, across all samples in the dataset [33,34]. The Pielou index is a way to measure even distribution of species (ASVs) in a population. It varies between 0 and 1, 1 representing a community with perfect evenness, and it decreases to 0 as the relative abundances of the species (ASVs) diverge from evenness. Faith’s PD is also provided, which accounts for species evenness in addition to species richness. Phylogenetic diversity (PD) is a measure of biodiversity based on phylogeny. Faith defined the phylogenetic diversity of a set of species (ASV) as equal to the sum of the lengths of all those branches on the tree that span the members of the set [35,36].

**Beta diversity** indices were also calculated. Jaccard and Bray–Curtis similarity indices were performed to compare different communities from different sites. The Bray–Curtis similarity is based on occurrence data (abundance), while the Jaccard distance is based only on presence/absence data.

The Bray–Curtis similarity index compares the relative abundances of a community across two sites. A value of 1 indicates total similarity, and a value of 0 indicates total dissimilarity. Weighted UniFrac, which accounts for abundance of observed organisms’ distances among sites and unweighted (qualitative), which considers only their presence or absence, were also computed.

## 3. Results

### 3.1. Organic Matter, pH, and Total Soil Metal Content

**Figure 2:**
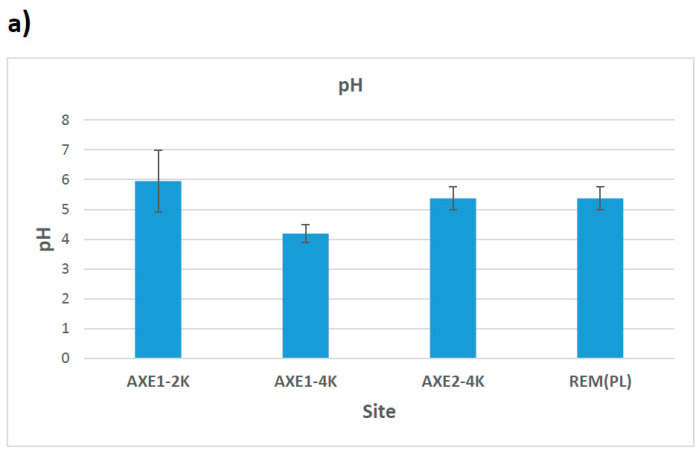

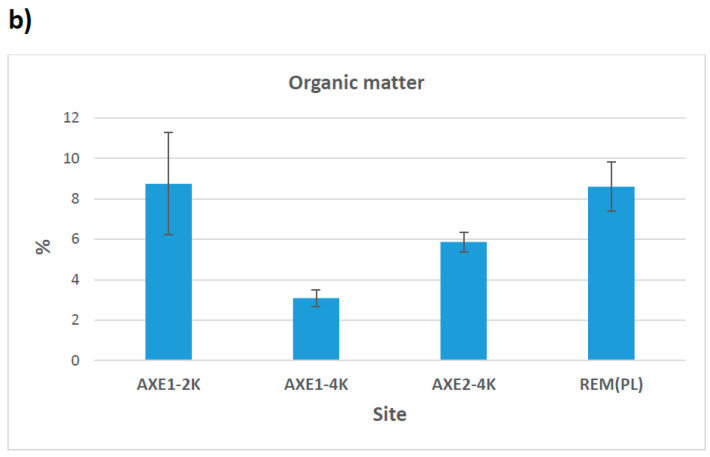
Means levels of (**a**) pH and (**b**) organic matters at each of the targeted sites, Axe1-2k: site 1 (residential area); Axe1-4k: site 2 (dry agricultural land); Axe-4k: site 3 (wet agricultural field); and Rem: the remediated tailing (site 4). Bars represent standard errors.

The soil pH was acidic, varying between 4.2 and 6 (Figure 2). The highest levels of pH were found at the residential site 1 (pH = 6) and the lowest at the agricultural dry land (site 2) with a pH of 4.2. The agricultural wetland and the remediated tailing (site 4) both showed a similar pH level of 5.4. The highest levels of organic matter (8.3%) were observed at the residential site and the remediated tailing. The lowest amount of OM (2.5%) was observed in the agricultural dry land samples (site 2). The level of OM in samples from site 3 was 5.9% (Figure 2). The pH, organic matter content, and metals in soil from the rhizosphere and non-rhizosphere in the tailing site were not statistically different. Hence, for comparison purposes with other sites, all the soil analyses focused only on non-rhizospheric soil with the exception of Cu/Co translocation in plants growing in the tailing.

The levels of copper, cobalt, magnesium, and manganese are presented in Table 1. The highest content of total Cu and Co were found at the remediated tailing, with soil concentrations of 9445 mg/kg and 2228 mg/kg, respectively. This was followed by the residential site, with 579 mg/kg of Cu and 110 mg/kg of Co. The levels of these metals in both dry and wet agricultural fields were significantly low. In fact, there was 228 times more total Cu in the remediation tailing site (9447 mg/kg) compared with the agricultural dry land and 495 and 16 times more compared with samples from the agricultural wetland and residential areas, respectively. Similarly, the level of total Co was 500 times more in the remediation tailing site than in the samples from the dry agricultural field and 80 and 20 times more compared with the agricultural wetland and residential areas, respectively. The remediated site also contained the highest content of magnesium and manganese. Interestingly, as with Cu and Co, the levels of Mg and Mn were higher in the residential areas compared with the agricultural dry and wet fields. The total amount of Al was higher in the remediated tailing site followed by the residential and agricultural wet areas. The lowest level of Al was observed in the agricultural dry site. For Fe, the highest soil concentration was found in the residential and remediated tailing sites followed by the dry and wet agricultural areas. The bioavailable levels of all the metals measured (Cu, Co, Mg, and Mn,) at all the targeted sites were below 5 mg/kg.

### 3.2. Bioaccumulation, and Translocation Factors

**Figure 3:**
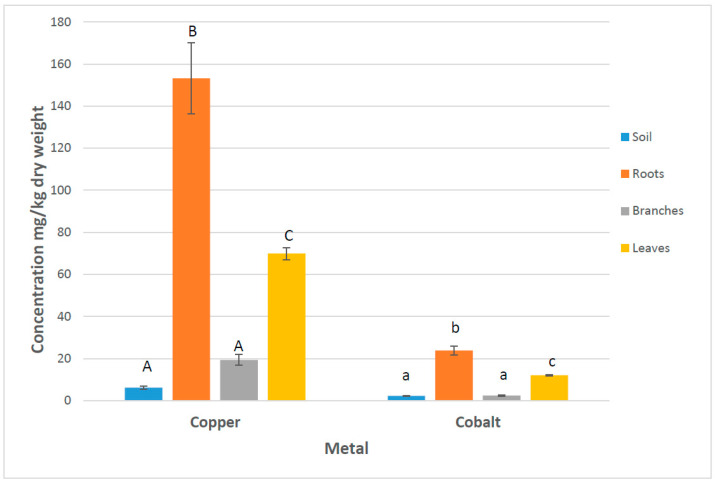
Bioavailable metal concentration in soil rhizosphere and total metal concentration in *Leucaena leucocephala* roots, branches, and leaves growing in remediated tailing (site 4). Means above error bars with different letters for each metal (copper and cobalt) are significantly different (*p* ≤ 0.05). Bars represent standard errors. Means with different letters for each metal (copper and cobalt) are significantly different (*p* ≤ 0.05).

The bioaccumulation factor (BF) represents the proportion of metals in roots over the bioavailable metals in the soil from the plant rhizosphere. These values were calculated only for the *Leucaena leucocephala* that were planted on the remediation tailing site for phytoextraction purposes. The levels of Cu and Co in soil and *L. leucocephala* tissues are described in Figure 3. The BF based on the bioavailable Cu in the soil was 32.23. These values were 0.021 when the soil total Cu was considered. The BF was 11.3 when the soil bioavailable Co was used and 0.011 when the soil total values were considered.

The translocation factors (TFs) that reflect the mobility of metals from roots to branches were 0.13 for Cu and 0.18 for Co. The TF values from roots to leaves were 0.45 for both Cu and Co, indicating that *L. leucocephala* is a Cu/Co excluder.

### 3.3. Microbial Analysis

**Figure 4:**
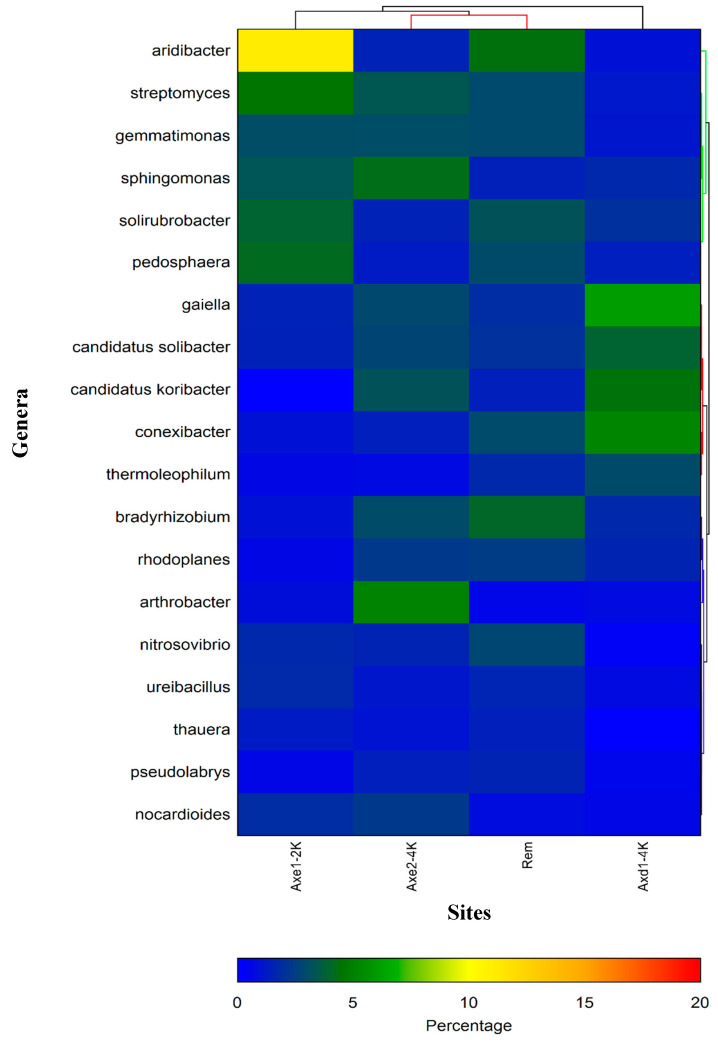
Bacterial communities: dual hierarchal dendrogram evaluation of the taxonomic classification data with each sample clustered on the X-axis labeled based upon the treatment. Samples with more similar microbial populations are mathematically clustered closer together. The genera (consortia) are used for clustering. Thus, the samples with more similar consortia of genera cluster closer together with the length of the connecting lines (top of heatmap) related to the similarity; shorter lines between two samples indicate closely matched microbial consortia. The heatmap represents the relative percentages of each genus. The predominant genera are represented along the right Y-axis. The legend for the heatmap is provided in the bottom, representing the relative abundance (percentage of each genus). Axe1–2K represents site 1 (residential area); Axe1–4K or Axd1-4 represents site 2 (dry agricultural land); Axe2–4K represents site 3 (wet agricultural field); and Rem represents remediated tailing (site 4).

**Figure 5:**
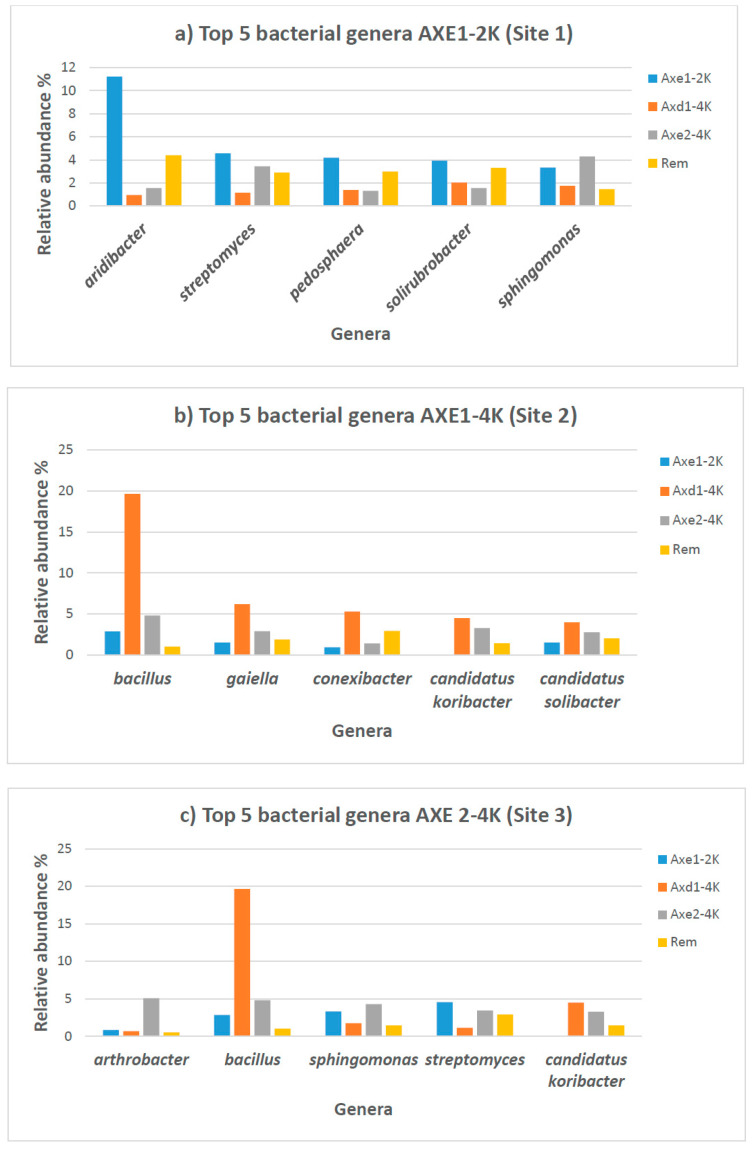

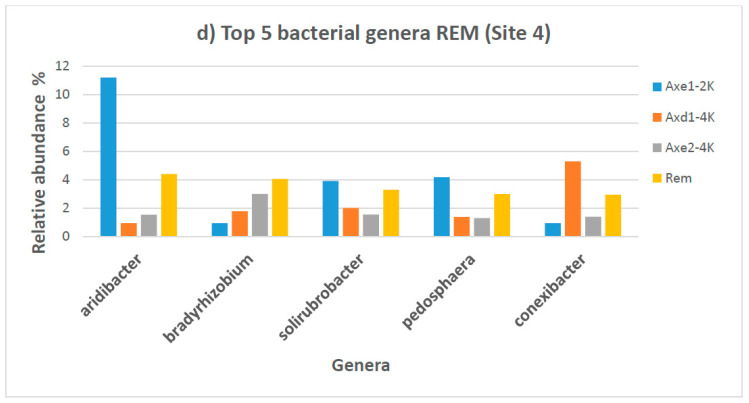
Top five bacterial genera and their relative abundance in soil samples from each site along with other sites: (**a**) Axe1-2K (site—1: residential area); (**b**) Axe1-4k or Axd1-4K (site 2: agricultural dry land); (**c**) Axe2-4K (site 3: agricultural wetland); and (**d**) Rem (remediated tailing) (site 4). Data in percentages.

**Figure 6:**
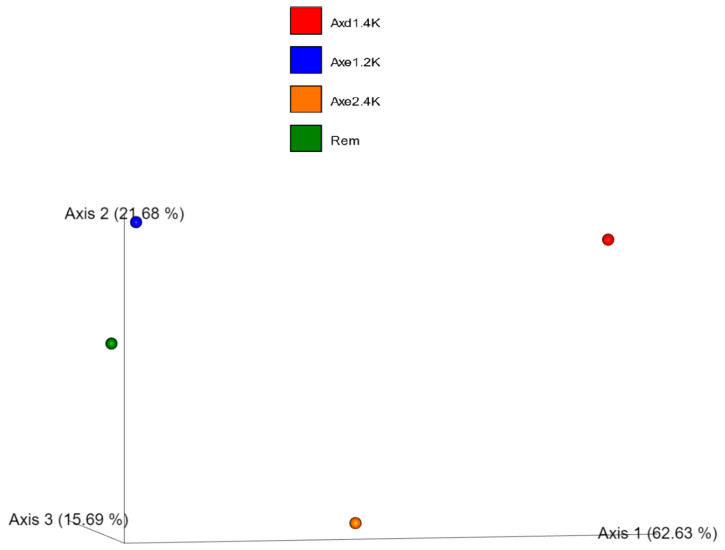
Principal coordinate plot of weighted UniFrac data for bacterial communities. Axe1–2k represents site 1 (residential area); Axe1–4k or exd1-4k: site 2 (agricultural dry land); Axe2–4k: site 3 (agricultural wetland); and Rem: remediated tailing (site 4). The plot was generated using amplicon sequence variant (ASV) data.

**Figure 7:**
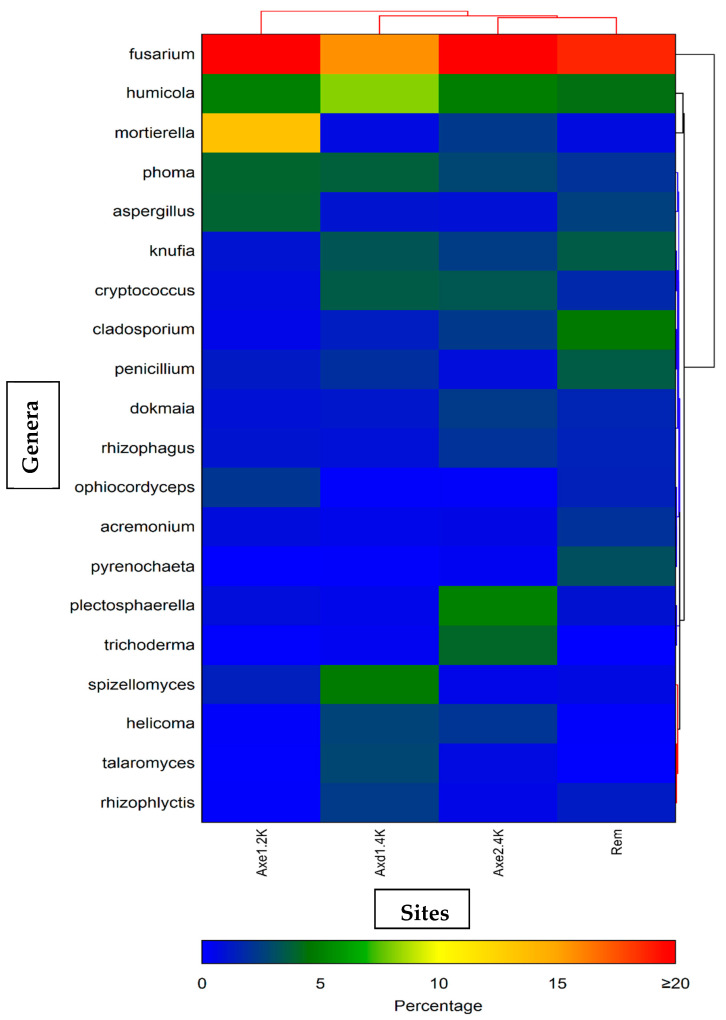
Fungal communities: dual hierarchal dendrogram evaluation of the taxonomic classification data with each sample clustered on the X-axis labeled based upon the treatment. Samples with more similar microbial populations are mathematically clustered closer together. The genera (consortia) are used for clustering. Thus, the samples with more similar consortia of genera cluster closer together, with the length of the connecting lines (top of heatmap) related to the similarity; shorter lines between two samples indicate closely matched microbial consortia. The heatmap represents the relative percentages of each genus. The predominant genera are represented along the right Y-axis. The legend for the heatmap is provided at the bottom, representing relative abundance (percentage of each genus). Axe1–2k represents site 1 (residential area); Axe1–4k or Axd1-4k represents site 2 (dry agricultural land); Axe2–4k represents site 3 (wet agricultural field); and Rem represents remediated tailing (site 4).

**Figure 8:**
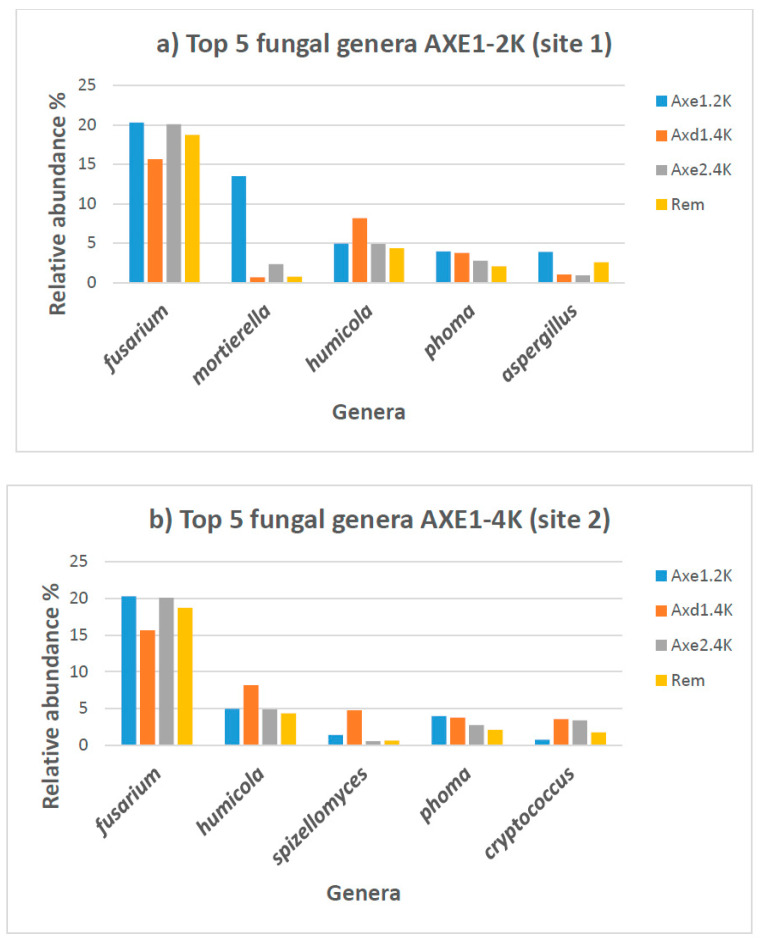

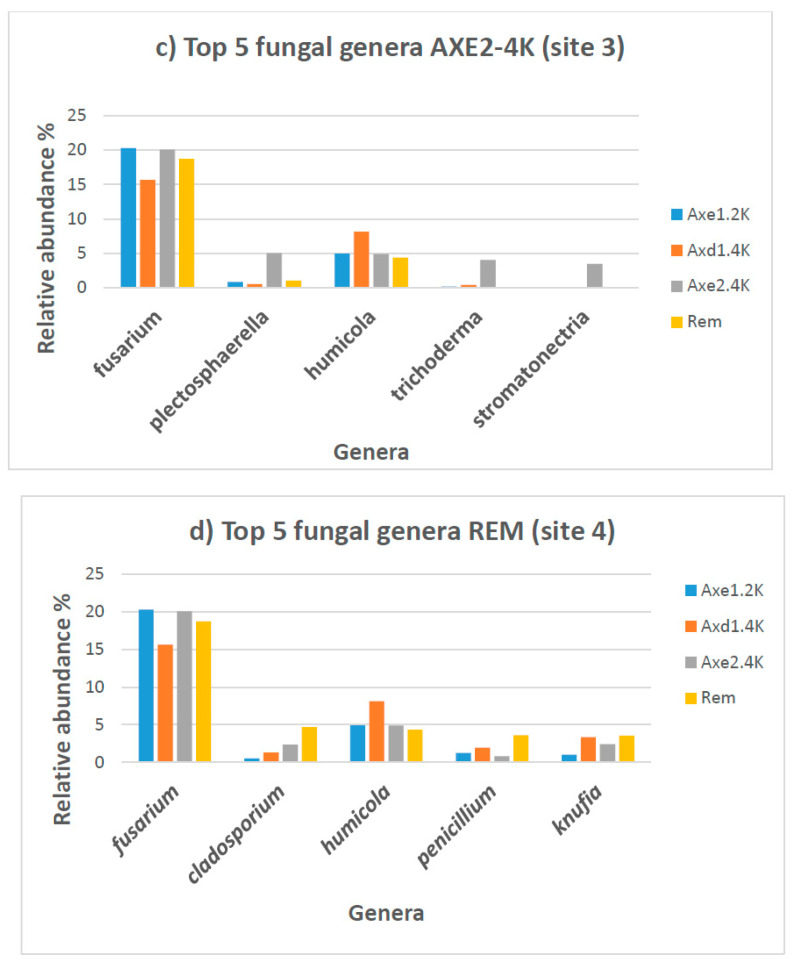
Top five fungal genera and their relative abundance in soil samples from each site along with other sites: (**a**) Axe1–2k: site 1 (residential area); (**b**) axd1-4k or Axe1-4k: site 2 (agricultural dry land); (**c**) Axe2-4k: site 3 (agricultural wet field); and (**d**) Rem: remediated tailing (site 4). Data in percentage.

The reads generated in this project have been deposited in the NCBI Short Read Archive database (BioProject accession number: PRJNA1154301). Overall, 3036 unique zOTUs were identified. Variable numbers of zOTUs were recorded at each site, including Axe1–4 k (1996 zOTUs), Axe1–2 k (1987 zOTUs), Axe2–4 k (2489 zOTUs), and remediation site (2302 zOTUs). For bacteria, the number of phyla identified in the 4 sites analyzed varied from 13 to 17, 38 to 45 for classes, 168 to 190 for families, and 326 to 389 for genera. For fungi, there were 8 phyla identified in each of the 4 sites, 27 to 31 classes, 189 to 225 families, and 335 to 427 genera in the samples from the targeted sites. Table S1 reports the numbers of families, classes, and phyla. Details of the bacterial and fungal abundance and alpha and beta diversity measures are described in Table 2, Table 3, Table 4, Table 5, Table 6, Table 7, Table 8 and Table 9, Figure 4, Figure 5, Figure 6, Figure 7 and Figure 8, as well as in Tables S1–S7 and Figures S1–S5.

#### 3.3.1. Bacterial Populations

A total of over 379,000 sequence pairs were parsed, and ASVs for QIIME 2 analysis, that used an average of over 454,000 sequences per sample, were successfully mapped to ASVs. For secondary taxonomic analysis using zOTUs, over 126,000 merged paired sequences were mapped within the Bacteria domains that were utilized for final taxonomic analyses. In QIIME 2 for alpha and beta diversity analysis, samples were rarefied to 30,000 per sample for feature ASV analysis.

##### Relative Abundance 

Table 2 and Table S7 list all the genera with their relative abundance in each sample. There was a wide range of top 40 genera found to be significantly different among groups (Table 2). For example, *Bacillus* exhibits 19.3% of the total bacteria in site 2 (agricultural dry field), while in samples from the tailing site, this genus represented only 1.02% of the bacterial population. The heatmap represents the relative percentages of each genus. Overall, the most abundant bacterial genera at the residential site include *Acidibacter* (11.1%), *Pedosphaera* (4.2%), Streptomyces (4.5%), *Solirubrobacter* (3.9%), and *Sphingomonas* (3.3%). *Bacillus*, with a relative abundance of 19.3%, was the most preponderant genus in the dry agricultural area (site 2), followed by *Gaiella* with 6.1%, *Conexibacter* with 5.2%, *Candidatus Koribacter* with 4.4%, and *Thermoleophilum* with 3%. A different bacterial profile was observed at the agricultural wet site, where *Bacillus* was the dominant genus, with 4.8% of all genera, followed by *Sphingomonas* with 4.3%, *Streptomyces* with 3.4%, *Candidatus Koribacter* with 3.3%, and *Gemmatimonas* with 3%. *Aridibacter* was the most predominant genus in the tailing, representing 4.4% of the total bacterial population, followed by *Bradyrhizobium* at 4.3%, *Solirubrobacter* at 3.3%, *Pedosphaera* at 3%, and *Gemmatimonas* at 2.9%.

The Pearson correlations were calculated based on genera counts. The coefficient between site 1 and site 2 was only 0.09; 0.27 for site 1 vs. site 3; 0.65 for site 1 (1–2 km) vs. site 4 (remediated tailing); 0.51 for site 2 vs. site 3; 0.05 for site 2 vs. site 4 (remediated tailing); and 0.28 for site 3 vs. site 4 (remediated tailing).

To provide a visual overview combined with analysis, we utilize a dual hierarchal dendrogram to display the data for the top 30 most predominant genera with clustering related to the different groups. Three primary groupings are shown among the samples, as seen on the top of the dendrogram in red illustrating the two groups (agricultural dry area and remediated tailing) with the closest similarity based on bacterial relative abundance (Figure 4). The top five bacterial genera for each site are described in Figure 5.

##### Unique Genera

For bacteria, the unique genera at site 1 (residential area) included *Geofilum, Rhodothalassium*, and *Gulbenkiania;* at site 2 (dry agricultural site) *Caldalkalibacillus, Ferritrophicum, Planifilum, Acetomicrobium, Crinalium*, and *Annamia;* at site 3 (wet agricultural area) *Castellaniella, Pedobacter, Janthinobacterium*, and *Curvibacter;* at the phytoremediation site *Shewanella, Segetibacter, Thermotoga, Oceanobacillus, Thermosiphon, Kallotenue*, and *Ardenticatena* were unique.

##### Alpha Diversity

The Observed Features (ASV) chart illustrates that a plateau was achieved for each group. The most observed features (ASV), with 878 counts, were found in samples from site 1 (residential area) and the least, with 585 ASV counts were samples from the agricultural wetland (Figure S1).

The Shannon diversity entropy (SDE) showed strong similarity among the four sites, with the populations from site 1 (residential area) and site 4 (remediated tailing) showing the most similar Shannon entropy values (Table 3 and Figure S2). The Shannon diversity entropy values for site 2 (dry agricultural area) and site 3 (wet agricultural site) were also almost identical. Pielou species evenness, which measures how evenly the species are distributed in a community, showed that all four communities were similar, with values ranging from 0.89 to 0.94 on a scale of 0 to 1. However, the Faith PDs were different when the targeted sites were compared, with values ranging from 52 for site 3 to 76 for samples from site 1 (Table 3).

##### Beta Diversity

The microbial community structure was analyzed using weighted UniFrac distance matrix. Principal coordinate analysis plots were used to visualize the data in this distance matrix. Both Jaccard similarly and Bray–Curtis similarity distance showed strong similarity among the sites, with distance values ranging from 0.91 to 0.98 (Jaccard similarity) and 0.79 to 0.94 (Bray–Curtis similarity) (Table 4 and Table S2).

The distance matrix values based on unweighted variants were high (values varying from 0.73 to 0.81). But the distance matrix values for the unweighted variants were low, varying between 0.21 and 0.36, showing closeness among bacterial communities based on presence/absence (Table 5 and Table S3). The principal coordinate plot of weighted UniFrac data are illustrated in Figure 6. It confirms the closeness between site 1 (residential area) and site 4 (remediated tailing) based on the relative abundance of ASV.

#### 3.3.2. Fungal Population

A total of over 1,500,000 sequence pairs were parsed, and ASVs for QIIME 2 analysis that used an average of over 606,000 sequences per sample were successfully mapped to ASVs. For secondary taxonomic analysis using zOTUs, over 555,603 merged paired sequences that were mapped within the fungal domains were utilized for final taxonomic analysis. In QIIME 2 for alpha and beta diversity analysis, samples were rarefied to 30,000 per sample for feature ASV analysis.

##### Relative Abundance

Table 4 and Table S8 list each genus with its relative abundance in each sample. It shows the top 40 genera across all samples. There was a wide range of genera found to be significantly different among the groups (Table 6). The heatmap represents the relative percentages of each genus (Figure 7). *Fusarium* exhibited the highest proportion of the fungal genera at all the sites, with a relative abundance ranging from 15.6% to 20.3%. In samples from site 1 (residential area), the proportion of *Fusarium* was estimated at 20.3%. *Mortierella* (13.5%) was the second most dominant genus, followed by *Humicola* (5%), *Phoma* (4%), and *Aspergillus* (3.9%). At site 2 (agricultural dry land), *Fusarium* accounted for 15.6%, and *Humicola*, with a relative abundance of 8.2%, was again the second most preponderant genus. *Spizellomyces* with 4.8%, *Phoma* with 3.8%, and *Cryptococcus* with 3.6% were also among the top five genera in this site. A slightly similar trend was observed for site 3 (agricultural wet land), with *Fusarium* representing 20% of all genera, followed by *Plectosphaerella* with 5%, *Humicola* with 4.9%, and *Cryptococcus* with 3.4%. *Fusarium* represented 18.7% of all the genera in the tailing site, followed by *Cladosporium* at 4.3%, *Humicola* at 3.5%, *Penicillium* at 3.3%, and *Knufia* at 3% (Table 6). Figure 8 shows the top five fungal genera at each site.

Unlike for bacteria, the correlation coefficients based on relative abundance showed that the four sites analyzed were closely related. The correlation coefficients were 0.70, 0.79, 0.75, 0.82, 0.79, and 0.85 for site 1 (residential area) vs. site 2 (agricultural dry area), site 1 (residential area) vs. site 3 (agricultural wetland), site 1 (residential area) vs. site 4 (remediated tailing); site 2 vs. site 3; site 2 vs. site 4; and site 3 (agricultural wetland) vs. site 4 (remediated tailing), respectively. 

##### Unique Genera

For fungi, *Saccharata, Rhabdocline*, and *Nalanthamala* were unique to site 1 (residential area); *Hyphodermella* and *Verpa* specific to site 2 (agricultural dry land); *Oligoporus* and *Clitocella* to site 3 (agricultural wet land); and *Parastagonospora* to the site 4 (remediated tailing).

##### Alpha Diversity

Like with bacteria, the observed features (ASV) chart illustrates that a plateau was achieved for each sample (Figure S3). Shannon diversity entropy indices were similar among the sites, varying between 7.0 and 7.4 (Table 7 and Figure S4). Likewise, Pielou evenness values were not significantly different and ranged from 0.79 to 0.81 (Table 7). Both of these indices were high, but lower compared with the bacterial values. However, the Faith PDs were significantly different when the targeted sites were compared, with values from 85 for the remediation site to 125 for samples from site 2 (agricultural dry land) and site 3 (agricultural wetland) (Table 7).

##### Beta Diversity

Like with the bacterial population, both Jaccard similarly and Bray–Curtis similarity distance matrices were high, with distance values ranging from 0.82 to 0.98 (Jaccard similarity) and 0.76 to 0.99 (Bray–Curtis similarity) (Table 8 and Table S4).

Distance matrix values based on unweighted variants were lower than in bacterial populations, with values varying between 0.61 and 72 (Table S5). But the distance matrix values for the weighted variants were intermediate, varying between 0.43 and 0.55 (Table 9 and Figure S5).

## 4. Discussion

### 4.1. Soil Physico-Chemistry

The effects of land use conversion on soil physicochemical properties have been well investigated in a number of studies [37,38] and are reported to vary across land use types [39,40]. This can alter soil quality by changing soil texture and pH, cycles of carbon and nitrogen, and soil organic matter (SOM) [41,42]. The levels of organic matter were the same in the residential area and the remediated tailing samples. Low levels of OM were observed in the agricultural areas. In fact, anthropogenic activities often affect the amount of organic matter in the soil. Cropping aerates the soil and increases the availability of stored carbon, resulting in an increase in microbial activity and, therefore, rates of decomposition. Hijbeek et al. [43] demonstrated that a deficiency of SOM is related to soil texture, slope, aridity, and land use. In the present study, the levels of pH were correlated with the OM content; high values were observed in the residential and tailing samples, with the lowest in agricultural dry land. Soil pH influences metal-solution and soil-surface chemistry. In general, at low pH, the metal adsorption is small. Adsorption then increases at intermediate pH from near zero to near complete adsorption over a relatively small pH range [44].

The highest levels of metals were found in the remediated tailing followed by the residential area. The dry and wet agricultural areas showed significantly low levels of Cu, Co, Mg, Mn, and Fe. The usually high concentrations of iron and aluminum in all the sites appear to be of natural origin. In fact, metals occur naturally in soils, sediments, rocks, and water in mining regions to the levels to be classified as “contaminated sites” [45,46]. The retention of metals depends on soil type and composition.

This study focuses on environmental risk assessment based on total metals in the soil and the proportion that is available to biota for uptake from the soil. The low level of bioavailable metals in this study is consistent with previous analyses of soil samples from the same region. Tembo et al. [47] reported low to moderate levels of bioavailable Cu, ranging from 0.20 to 58.2 mg kg^−1^, in soils from the mining town of Kabwe, a nearby copper belt city in Zambia, but much higher bioavailable levels of lead and zinc. On the other hand, Narendrula et al. [46], studying soils from the Etoile Mine in Lubumbashi, detected very low levels of bioavailable copper, varying from 0.27 to 1.8 mg/kg. Zhen et al. [7] demonstrated that both total and bioavailable metals can impact soil microbial communities.

### 4.2. Metal Accumulation and Translocation in Leucaena leucocephala

Plants can restrict metals from soils by restricting their entry into root cells. They can also prevent them from reaching active metabolic locations once inside the cell [48,49]. Plants also possess homeostatic cellular mechanisms to control the uptake, accumulation, and detoxification of metals [49,50,51]. The present study and previous investigations showed that the bioavailable levels of Cu and Co at several sites within the City of Lubumbashi are low. Hence, the high levels of Cu in *Amaranthus vulgarus*, *Brassica chinensis*, *Spinacia oleracea*, and *Brassica carinata* reported by Mununga Katebe et al. [52] suggests that these species are Cu accumulators [48]. Malaisse and Gregoire [53] reported preliminary data on levels of copper in plants growing on the Etoile mining site. They showed that *Acalypha cupricola* presents higher copper contents in its roots than in its leaves, an indication of a Cu exclusion mechanism. Detailed analysis of Cu and Co movement in *Leucaena leucocephala* used for phytoremediation of the tailing in this study revealed that this species is also a Cu or Co excluder. In fact, it does store Co in roots without accumulation in aerial parts. Being a leguminous species with nitrogen fixing capability and resistant to Cu and Co, *L. leucocephala* could be an excellent species for the reclamation of the targeted tailing. Its phytoextraction potential to remove Co or Cu in soils can contribute to the sustainability of the site.

Considering that this study is the first documented report on the response of *Leucaena leucocephala* to Cu and Co contamination, analysis at several other sites is warranted to confirm these results. Investigations on other species used for phytoremediation in Cu and Co contamination are also needed.

### 4.3. Response of Microbial Communities to Environmental Stressors

Microorganisms have been shown to rapidly respond to soil environmental changes [54,55]. Singh et al. [56] investigated the impact of metals on soil microorganisms using 454 pyrosequencing platforms in two grassland plots contaminated with up to 200 mg/kg of Cu and 450 mg/kg of zinc. They reported a significant decline in microbial diversity and function associated with long-term exposure to metal stresses. Likewise, Chen et al. [57] used Denaturing Gradient Gel Electrophoresis (DGGE) to determine that Cd, Cu, Pb, and Zn pollution resulted in a decrease in soil microbial abundance, diversity, and activity. Chodak et al. [58] also showed that high levels of Cu, Zn, and Pb in soil negatively affected the Chao1 diversity index without changes in microbial structure.

On the other hand, Hong et al. [59] showed using Illumina MiSeq sequencing that metal-impacted soil had a significantly higher bacterial alpha diversity compared with unpolluted soils. Detailed examination of Hong et al. [59]’s data showed that the levels of metal content in the soil were rather low, within acceptable contamination levels by several guidelines. Berg et al. [60] investigated bacterial community composition and diversity along a Cu gradient (20 to 3537 mg/kg) in soils contaminated with CuSO_4_ for more than 85 years. They found no significant correlation between bacterial OTU richness and bioavailable Cu based on tag-coded pyrosequencing analysis.

In this study, the Illumina MiSeq platform was used to analyze, for the first time, bacterial and fungal dynamics and structures. It should be pointed out that Illumina sequencing processes at a high level of resolution compared with the older methods such as 454 used so far to study microbial community diversity and structure [61]. This platform utilizes a sequencing-by-synthesis (SBS) approach. This is widely used because of its scalability, high yield, large throughput, and short run time [61,62].

There were significant variations in the relative abundance of the top 20 genera, but the most prevalent genera were somewhat similar at each site regardless of the ecological properties. For example, cluster analysis revealed that site 3 (agricultural wetland), with low levels of Cu and insignificant levels of Co associated with low OM, and site 4 (remediated tailing), with high levels of both Cu and Co and high OM content, were the most closely related based on bacterial abundance. For fungi, the genus *Fusarium* was the most abundant in all the sites, representing about 16% to 20% of all fungi at each area regardless of Cu and Co content. A recent analysis showed that both total and bioavailable metals can impact microbial abundance and structures. Hence, although the number of sites was limited, there was no link between the level of organic matter, total Cu and Co, and bacterial and fungal abundance.

Soil pH has been classified as the key factor shaping the soil microbial populations [7,63]. Even a small change in pH affects single-celled organisms, as the intracellular pH is usually within 1 pH unit of neutral in most microorganisms [7,64]. Nkongolo et al. [9,10] showed that ameliorating soil acidity by liming plays a significant role in microbial community composition and relative abundance. Interestingly, in this study the sites that were the most closely related based on the relative abundance of genera (Axe2–4 k and remediated tailing) had identical pH values (pH = 5.3). The least acidic site (residential site) and the most acidic (agricultural dry land) showed similar levels of microbial abundance. Hence, the pH did not play a significant role in the level of microbial abundance. Bacterial and fungal variations in composition and relative abundance were observed among the sites but with no discernible link with pH changes.

It should be noted that in this study, zOTUs were used instead of OTUs. Traditional OTUs are generated by a cluster of sequencing reads that have, in most cases, >97% similarity to one another. A key reason many researchers are prompted to use zOTUs (zero-radius OTUs) or ASVs (amplicon sequence variants) is the loss of biological information. In the case of zOTUs, all correct biological sequences are identified, distinguishing sequences with even a single difference. This level of specificity is not achievable when using a 97% identity threshold as with traditional OTUs, and as a result, different strains or species with closely matching sequences are clustered together. Using zOTUs over traditional OTUs provides a greater resolution of all biological sequences. The number of zOTUs generated in samples from Axe1–2 k (1996) and Axe1–4 k (1987) were almost identical. Likewise, zOTUs counts in samples from Axe2–4 k and the tailing sites were similar (2489 and 2302, respectively), following the same trend as the clustering analysis.

The alpha diversity indices (Shannon entropy and Pielou evenness) for the four sites analyzed were similar for the bacterial populations. The same trend was observed for fungal communities, but the values were slightly lower. This indicates that the levels of bacterial and fungal diversity within each site were high and similar regardless of significant differences in Co and Cu content among the sites, especially between the remediated tailing and other areas. Likewise, the Pielou index data confirmed that the evenness indices based on ASV within each bacterial and fungal community were similar.

The beta similarity indices also showed that the four populations are closely related based on the Bray–Curtis distance matrix (using abundance data) and Jaccard’s distance (using presence/absence data) matrices for both bacteria and fungi. Unlike the Jaccard’s and Bray–Curtis distance matrices, UniFrac uses phylogenetic information to compare samples from the environment. It is an effective distance metric for microbial community comparison [65]. The results of the present study revealed that the bacterial and fungal communities were somewhat distant from each either (values ranging from 0.73 to 0.83 for bacteria and from 0.61 to 0.72 for fungi) when unweighted UniFrac analysis (based on presence/absence) was used. However, the weighted UniFrac data (based on abundance) showed that the communities were from close to intermediate (values ranging from 0.20 to 0.36 for bacteria and from 0.43 to 0.55 for fungi). Weighted UniFrac is useful for assessing differences in microbial community structures, uses abundance to calculate distance, and the effect of low abundance features is reduced. Weighted UniFrac does not consider the variation in the weights under random sampling, resulting in less power in detecting the differences between communities [66]. On the other hand, the unweighted UniFrac is more sensitive to differences in low-abundance features [66].

### 4.4. Adaptation of Bacterial and Fungal Communities to Environmental Stressors

The development of second- and third-generation sequencing technologies has provided unique insights into the adaptation strategies used by microorganisms to deal with metal contamination [20]. It is documented that either total or bioavailable metals play important roles in structuring microbial communities [63,67]. This is supported by Zhen et al. [7], who reported that both the total amount and availability of metals influence significantly the soil microbial community and ecology across land use patterns. Hence, they both should be used simultaneously for risk assessment of ecosystems.

The results of the present study suggest that bacterial and fungal communities in the targeted sites in the City of Lubumbashi have adapted to high level of soil contamination, especially in the remediated tailing (9446 mg/kg of Cu and 2228 mg/kg of Co). Considering that the exploitation of the metal deposits began several decades ago, the lack of differences in the microbial community abundance and diversity between the highly contaminated tailing and the less contaminated sites observed in the present study suggest resilience and/or physiological adaptation.

Li et al. [68] and Bourceret et al. [69] demonstrated that microbial communities do adapt to high levels of metal contamination. They showed that exposure for five years to copper stress in soils artificially polluted with 3200 mg of Cu per 1 kg of soil increased the resistance of soil microbial communities to subsequent copper stress. Bourceret et al. [69] reported that in industrial wasteland soils contaminated with Cr, Cu, Zn, and Pb, microbial species richness and diversity remained high, an indication of the long-term adaptation and resilience of the microbial communities changing to a diversified and metal-resistant community. Gołebiewski et al. [70], using pyrosequencing, found that in spite of the very high Pb and Zn concentrations in soil, the abundance of the levels of bacterial species richness, diversity, and evenness were similar in all samples, irrespective of the metal content levels.

This adaptation process was confirmed by Nkongolo et al. [9,10]. In fact, they reported no significant differences in different microbial diversity indices among the bacterial communities from different tailing areas contaminated with different levels of nickel, copper, and other metals in the City of Greater Sudbury (CGS) (Canada) using amplicon Illumina sequencing [9,10]. Li et al. [71] used a similar approach of high-throughput sequencing to analyze soil microorganisms under five-year nickel pollution in China and observed no clear trend in the bacterial diversity and abundance. Other studies report that long-term metal exposure leads to alterations in the microbial composition within communities toward a higher level of physiological adaptation [72,73,74,75,76,77]. Details on the mechanisms of metal adaptation and resilience to metal pollutants are described in detail in Azarbad et al. [20].

### 4.5. Impacts of Microorganism Assemblages on Leucaena leucocephala’s Ability to Phytoremediate Metal-Contaminated Tailing Sites

The rhizosphere is an integral part of a plant’s living system. It is characterized by the exchanges between the roots and the soil components. This results in interdependence between the soil components and the root activity [78]. A number of exchanges occur in the rhizosphere between biotic and abiotic components such as fungi, bacteria, gases, and minerals [79]. Hence, a healthy rhizosphere with thriving communities of microbes and other biotic components is critical to plant growth and survival. Nkongolo et al. [21] revealed higher Chao1, Shannon index, and species richness in bacterial populations from phytoremediated areas compared with other sites.

Likewise, Yan et al. [80] observed an increase in microbial diversity and the abundances of Proteobacteria and Bacteroidetes in soil rhizospheres contaminated with metals. They reported negative Pearson correlations between the levels of mercury, arsenic, nickel, chrome, and copper and Proteobacteria (*p* < 0.05). Their findings support that using suitable local plants is a promising approach for repairing heavy metal contaminated coastal soil.

Li et al. [81] showed that Cr and Mn negatively affect soil microbial α-diversity. Yunhua et al. [82] showed that some rhizosphere bacterial taxa (e.g., *Actinomarinicola, Bacillariophyta*, and *Oscillochloris*) affected the levels of organic matter and pH of the rhizosphere soil. They also increased the mobility of metals in plants. Zhao et al. [83] demonstrated that microbial communities in soil were mainly affected by metal pollution, pH, and above-ground vegetation. Their study demonstrated that soil pH significantly influences the abundance and structure of most microorganisms.

In the present study, the levels of total Cu and Co in the rhizosphere available to biota, including microorganisms, was low, limiting their impact on microbial abundance and structure. The accumulation in roots was high, but their translocation in aerial parts of the plants very low, meaning that *Leucaena leucocephala* is able to extract Cu and Co from soil without impairing its growth.

## 5. Conclusions

This study revealed significant differences among sites based on soil physico-chemistry and ecological properties that are associated with the land’s use. It revealed also that *Leucaena leucocephala*, one of the main species grown on metal-contaminated lands, is a Cu/Co excluder and is suitable for the extraction of Cu and Co. Amplicon sequence analysis identified 3036 unique zOTUs, with their total number varying among the sites. Although there was a wide range of bacterial and fungal genera that were significantly different among the sites based on relative abundance, *Acidibacter* was the most abundant bacterial genus in the residential and remediated tailing sites at 11.1% and 4.4%, respectively, and *Bacillus* was predominant in both dry (19.3%) and wet (4.8%) agricultural lands. Surprisingly, the fungal genus *Fusarium* was the most predominant at all the sites, varying between 16% and 20% of all the fungi. The alpha diversity indices (Shannon entropy and Pielou evenness), which measured the diversity within each site, were similar for both the bacterial and fungal communities in all the sites. In fact, the Shannon diversity entropy index varied between 8.3 and 9 for bacteria and 7.0 and 7.4 for fungi. Likewise, Pielou evenness values ranged from 0.9 to 0.94 for bacteria and 0.79 to 0.81 for fungi. Beta diversity indices confirmed the closeness of the sites analyzed. Surprisingly, clustering analysis based on relative abundance and weighted UniFrac data revealed that site 2, located in a residential area at 2 km from the mining area, was more closely related to the remediated tailing, which contained the highest levels of Cu and Co. The lack of differences in microbial community diversity and structures between the highly contaminated and less contaminated sites observed in the present study suggest microbial resilience and physiological adaptation. Further studies should investigate other soil fauna such as eukaryotes, protozoans. etc. The metal extraction potential of other plant species also can be assessed.

## Figures and Tables

**Table 1:** Metal content at four sites in the cupriferous city of Lubumbashi.

	Al	Fe	Cu	Co	Mg	Mn
Sites			mg kg^−1^			
Site 1	11,666.67 ± 406 ^b^	31,833.33 ± 517 ^b^	579.00 ± 44 ^b^	110.47 ± 15 ^a^	1046 ± 77 ^b^	508.33 ± 48 ^a^
Site 2	5483.33 ± 337 ^a^	13,190 ± 2039 ^a^	41.27 ± 4 ^a^	4.44 ± 1 ^a^	176.33 ± 73 ^a^	113.13 ± 48 ^a^
Site 3	11,066.67 ± 328 ^b^	18,233.33 ± 393 ^a^	190.67 ± 24 ^a^	27.83 ± 2 ^a^	618.33 ± 51 ^a^	359.33 ± 11 ^a^
Tailing (Site 4)	17,966.67 ± 303 ^c^	32,133.33 ± 545 ^b^	9446.7 ± 192 ^c^	2228 ± 132 ^b^	9337 ± 247 ^c^	2693.67 ± 93 ^b^

Site 1: residential land. Site 2: agricultural dry land. Site 3: agricultural wetland. Site 4: remediated tailing. Means with different superscript letters are significantly different (*p* ≤ 0.05).

**Table 2:** Relative abundance (%) of the top 40 bacterial genera across all sites.

Genus	Site 1	Site 2	Site 3	Site 4
*Bacillus*	2.852169	19.30014	4.782069	1.019639
*Aridibacter*	11.10447	0.927474	1.531718	4.372573
*Gaiella*	1.506972	6.096894	2.869903	1.897923
*Streptomyces*	4.512124	1.123577	3.417418	2.885522
*Sphingomonas*	3.305843	1.710281	4.264329	1.443703
*Solirubrobacter*	3.896675	1.986755	1.538334	3.2832
*Conexibacter*	0.931966	5.214428	1.391117	2.930755
*Candidatus Solibacter*	1.501697	3.897962	2.742536	2.0242
*Gemmatimonas*	3.056147	1.07857	2.998925	2.904369
*Pedosphaera*	4.156922	1.356651	1.298486	2.960911
*Bradyrhizobium*	0.935483	1.756896	2.974113	4.033322
*Candidatus Koribacter*	0.070337	4.420369	3.258622	1.436164
*Arthrobacter*	0.837011	0.68797	5.053345	0.540917
*Rhodoplanes*	0.599624	1.64277	2.360433	2.41057
*Nitrosovibrio*	1.714467	0.268437	1.578033	2.755475
*Thermoleophilum*	0.643584	2.959236	0.653379	1.765992
*Nocardioides*	1.869208	0.596348	2.312464	0.742584
*Ureibacillus*	1.781287	0.715296	1.090067	1.590712
*Pseudolabrys*	0.564455	0.496689	1.412621	1.564326
*Thauera*	1.27486	0.139844	0.97097	1.439934
*Ktedonobacter*	0	2.668296	0.577289	0.533379
*Saccharopolyspora*	2.240236	0.355237	0.051278	0.978175
*Aciditerrimonas*	0.74909	0.877644	0.635183	1.174187
*Microvirga*	0.45895	0.707259	1.210818	1.010215
*Chthoniobacter*	0.13364	0.768341	0.570672	1.775416
*Blastococcus*	0.24618	0.649392	0.545861	1.343812
*Acidibacter*	0.599624	0.318267	0.573981	1.257115
*Mycobacterium*	0.645343	0.560985	0.646762	0.83682
*Skermanella*	0.891523	0.1736	0.271276	1.298579
*Steroidobacter*	1.223865	0.030541	0.395335	0.899016
*Nitrospira*	0.55918	0.281296	1.037135	0.523955
*Thermogemmatispora*	0	1.843696	0.30105	0.177165
*Terrimonas*	1.026922	0.056259	0.181954	1.036601
*Actinomadura*	0.014067	1.54954	0.567364	0.154548
*Gemmata*	0.43609	0.506333	0.325862	1.006446
*Hyphomicrobium*	0.423781	0.028933	1.156232	0.644578
*Geodermatophilus*	0.383337	0.896933	0.200149	0.761431
*Sphaerobacter*	0.571489	0.361667	0.873377	0.378831
*Reyranella*	0.453674	0.149489	0.750972	0.780278

Site 1: residential land. Site 2: agricultural dry land. Site 3: agricultural wetland. Site 4: remediated tailing.

**Table 3:** Alpha diversity for 16S (bacteria): Shannon entropy, Pielou evenness, Faith phylogenetic distance (PD), and observed amplicon sequences.

Sites	Shannon Entropy	Pielou Evenness	Faith PD	Observed Features (Amplicon Sequence Variants—ASVs)
Site 1	8.291692	0.891217	60.96604	632
Site 2	9.05744	0.925835	76.20122	881
Site 3	8.433922	0.917746	52.10697	584
Tailing (Site 4)	8.870255	0.942066	71.59701	683

Site 1: residential land. Site 2: agricultural dry land. Site 3: agricultural wetland. Site 4: remediated tailing. Faith PD is Faith’s phylogenetic distance.

**Table 4:** Beta measures for 16S (bacteria): Jaccard’s distance matrix.

	Site 1	Site 2	Site 3	Site 4
Site 1	–	0.974932	0.912343	0.958828
Site 2	0.974932	–	0.924376	0.912378
Site 3	0.912343	0.924376	–	0.921702
Site 4	0.958828	0.912378	0.921702	–

Site 1: residential land. Site 2: agricultural dry land. Site 3: agricultural wetland. Site 4: remediated tailing

**Table 5:** Beta measures for 16S (bacteria): Weighted UniFrac distance matrix.

	Site 1	Site 2	Site 3	Site 4
Site 1	–	0.363988	0.266722	0.206484
Site 2	0.363988	–	0.277756	0.351925
Site 3	0.266722	0.277756	–	0.240466
Site 4	0.206484	0.351925	0.240466	–

Site 1: residential land. Site 2: agricultural dry land. Site 3: agricultural wetland. Site 4: remediated tailing

**Table 6:** Relative abundance (%) of the top 40 fungal genera across all the sites.

Genus	Site 1	Site 2	Site 3	Tailing (Site 4)
*Fusarium*	20.29285	15.64247	20.07627	18.73673
*Humicola*	4.948659	8.155497	4.888755	4.354939
*Mortierella*	13.48544	0.685771	2.345615	0.733733
*Phoma*	3.954841	3.760141	2.757126	2.086299
*Knufia*	1.006391	3.34618	2.411456	3.560634
*Cryptococcus*	0.746018	3.565651	3.390853	1.757837
*Cladosporium*	0.546417	1.318012	2.351101	4.695267
*Aspergillus*	3.901404	1.03728	0.916299	2.588985
*Penicillium*	1.229044	1.94728	0.809306	3.601224
*Spizellomyces*	1.387783	4.754598	0.554169	0.65755
*Plectosphaerella*	0.823554	0.515666	4.987517	1.011615
*Dokmaia*	0.937762	1.103895	2.370305	1.603597
*Rhizophagus*	1.039396	0.893346	2.057557	1.471837
*Helicoma*	0.221605	2.712155	2.16455	0.163607
*Trichoderma*	0.132544	0.369353	4.035555	0.078057
*Rhizophlyctis*	0.202745	2.390384	0.581603	1.297615
*Ophiocordyceps*	2.232816	0.153451	0.222216	1.453104
*Acremonium*	0.773785	0.515666	0.636471	2.04571
*Talaromyces*	0.13202	2.779959	0.702313	0.139878
*Pyrenochaeta*	0.095348	0.158209	0.356643	3.132259
*Alternaria*	0.208508	0.248019	0.205756	3.011115
*Rhizoctonia*	1.272527	0.475817	0.318235	1.595479
*Epicoccum*	0.164501	1.387005	1.766755	0.237917
*Spiromastix*	0.117875	3.014298	0.260624	0.131135
*Stromatonectria*	0.016764	0.011301	3.445722	0.019983
*Leptosphaerulina*	0.137783	2.06326	0.614524	0.630698
*Cladorrhinum*	0.373533	0.735137	1.218074	1.113401
*Volutella*	0.272946	2.499227	0.441689	0.169851
*Exophiala*	0.275042	0.118359	1.492414	1.416885
*Clonostachys*	0.25461	1.551162	0.502044	0.494567
*Conocybe*	2.464376	0.060667	0.216729	0.052454
*Racocetra*	0.063391	0.073752	0.043895	2.386037
*Acrocalymma*	0.33005	0.093974	0.94922	1.178344
*Thanatephorus*	0.070725	2.162587	0.038408	0.160485
*Lectera*	0.132544	1.546404	0.570629	0.091795
*Dactylaria*	0.104254	0.061261	0.076815	2.082553
*Sordaria*	1.281957	0.602503	0.320979	0.095541
*Sarocladium*	0.175503	0.091595	0.101506	1.869614
*Chaetomium*	0.160834	0.88264	0.688596	0.417759

Site 1: residential land. Site 2: agricultural dry land. Site 3: agricultural wetland. Site 4: remediated tailing.

**Table 7:** Alpha measures for ITS (fungi): Shannon entropy, Pielou evenness, Faith phylogenetic distance (PD), and observed amplicon sequences.

Sites	Shannon Entropy	Pielou Evenness	Faith PD	Observed Features (Amplicon Sequence Variants—ASVs)
Site 1	7.071732	0.79423	94.07528	479
Site 2	7.376033	0.80682	124.6224	565
Site 3	7.444205	0.805789	124.8274	604
Site 4	7.031452	0.792966	84.87516	467

Site 1: residential land. Site 2: agricultural dry land. Site 3: agricultural wetland. Site 4: remediated tailing. Faith PD is Faith phylogenetic distance.

**Table 8:** Beta measures for ITS (fungi): Jaccard’s distance matrix.

	Site 1	Site 2	Site 3	Site 4
Site 1	–	0.859016	0.978302	0.818976
Site 2	0.859016	–	0.979039	0.840449
Site 3	0.978302	0.979039	–	0.974138
Site 4	0.818976	0.840449	0.974138	–

Site 1: residential land. Site 2: agricultural dry land. Site 3: agricultural wetland. Site 4: remediated tailing.

**Table 9:** Beta measures for ITS (fungi): Weighted distance matrix.

	Site 1	Site 2	Site 3	Site 4
Site 1	–	0.508031	0.486012	0.550006
Site 2	0.508031	–	0.432197	0.46487
Site 3	0.486012	0.432197	–	0.426722
Site 4	0.550006	0.46487	0.426722	–

Site 1: residential land. Site 2: agricultural dry land. Site 3: agricultural wetland. Site 4: remediated tailing.

## Data Availability

The reads generated in this project have been deposited in the NCBI Short Read Archive database (BioProject accession number: PRJNA1154301).

## Supplementary Materials

The following supporting information can be downloaded at: https://www.mdpi.com/article/10.3390/microorganisms13010031/s1, Table S1: Number of phyla, classes, families, and generation across targeted sites. Table S2: Bacteria Bray Curtis distance matrix. Table S3. Bacteria Unweighted UniFrac distance matrix. Table S4. Fungi—Bray Curtis distance matrix. Table S5. Fungi—Unweighted UniFrac distance matrix. Table S6. Bacterial genera count and relative abundance across sampling sites. Table S7. Fungal genera count and relative abundance across sampling sites. Figure S1. Observed Features (amplicon Sequence Variants) chart for bacterial communities. It shows that the plateau for each sample was achieved. The most observed features were found in sample from Axe-1-2K (878—ASV) and the least in samples from Axe2-4K (585 features—ASV). Axe1-2K: residential site; Axe1-4K or Axd1-4K: agricultural dry land, Axe2-4K: agricultural wet land, and REM: Remediated tailing. Figure S2. Shannon Diversity Entropy for bacterial communities showing the levels of diversity within each site based on Amplicon Sequences Variants. Axe1-2K: residential site; Axe1-4K or Axd1-4K: agricultural dry land, Axe2-4K: agricultural wet land, and REM: Remediated tailing. Figure S3. Observed Features chart for fungal communities that Illustrate that the plateau was achieved for each sample. The most observed features were found in sample Axe1-2K (878) (Axe4-605) and the least in sample Axe2-4K (585 features) (Axe1-2K (479). Axe1-2K: residential site; Axe1-4K or Axd1-4K: agricultural dry land, Axe2-4k: agricultural wet land, and REM: Remediated tailing. Figure S4: Shannon Diversity Entropy for fungal communities showing the levels of diversity within each Site. Axe1-2K: residential site; Axe1-4K or Axd1-4K: agricultural dry land, Axe2-4k: agricultural wet land, and REM: Remediated tailing. Figure S5. Principal coordinate plot of weighted UniFrac data for fungal communities Colors keyed on the treatment group. Primary vector explains 73% of the variation between the groups. The first three vectors together exhibit over 88% of the variation among the groups. Clear grouping of the samples can be observed (blue and green closest). (a) Axe1-2K: site 1 (residential area); (b) Axe1-4K or Axed1-4K: site 2 (agricultural dry land); (c) Axe2-4K: site 3 (agricultural wet field); and (d) Rem: Remediated tailing (site 4).

## References

[1] Turner B.L., Lambin E.F., Reenberg A. (2007). The Emergence of Land Change Science for Global Environmental Change and Sustainability. Proc. Natl. Acad. Sci. USA.

[2] Solar R.R.d.C., Barlow J., Andersen A.N., Schoereder J.H., Berenguer E., Ferreira J.N., Gardner T.A. (2016). Biodiversity Consequences of Land-Use Change and Forest Disturbance in the Amazon: A Multi-Scale Assessment Using Ant Communities. Biol. Conserv..

[3] Ferreira A.C.C., Leite L.F.C., de Araújo A.S.F., Eisenhauer N. (2016). Land-Use Type Effects on Soil Organic Carbon and Microbial Properties in a Semi-Arid Region of Northeast Brazil. L. Degrad. Dev..

[4] Baldrian P. (2017). Forest Microbiome: Diversity, Complexity and Dynamics. FEMS Microbiol. Rev..

[5] Guasconi D., Juhanson J., Clemmensen K.E., Cousins S.A.O., Hugelius G., Manzoni S., Roth N., Fransson P. (2023). Vegetation, Topography, and Soil Depth Drive Microbial Community Structure in Two Swedish Grasslands. FEMS Microbiol. Ecol..

[6] Mustafa G., Hussain S., Liu Y., Ali I., Liu J., Bano H. (2024). Microbiology of Wetlands and the Carbon Cycle in Coastal Wetland Mediated by Microorganisms. Sci. Total Environ..

[7] Zhen Z., Wang S., Luo S., Ren L., Liang Y., Yang R., Li Y., Zhang Y., Deng S., Zou L. (2019). Significant Impacts of Both Total Amount and Availability of Heavy Metals on the Functions and Assembly of Soil Microbial Communities in Different Land Use Patterns. Front. Microbiol..

[8] Briffa J., Sinagra E., Blundell R. (2020). Heavy Metal Pollution in the Environment and Their Toxicological Effects on Humans. Heliyon.

[9] Nkongolo K.K., Spiers G., Beckett P., Narendrula-Kotha R. (2022). Inside Old Reclaimed Mine Tailings in Northern Ontario, Canada: A Microbial Perspective. Ecol. Genet. Genom..

[10] Nkongolo K., Narendrula-Kotha R. (2023). Dynamic Changes of Microbial Community Composition and Diversity in Metal Contaminated and Reclaimed Lands Assessed by Illumina MiSeq Sequencing. Ecol. Genet. Genom..

[11] Lottermoser B.G. (2010). Mine Wastes (Third Edition): Characterization, Treatment and Environmental Impacts.

[12] Fashola M.O., Ngole-Jeme V.M., Babalola O.O. (2016). Heavy Metal Pollution from Gold Mines: Environmental Effects and Bacterial Strategies for Resistance. Int. J. Environ. Res. Public Health.

[13] Murty D., Kirschbaum M.U.F., Mcmurtrie R.E., Mcgilvray H. (2002). Does Conversion of Forest to Agricultural Land Change Soil Carbon and Nitrogen? A Review of the Literature. Glob. Chang. Biol..

[14] Meng M., Lin J., Guo X., Liu X., Wu J., Zhao Y., Zhang J. (2019). Impacts of Forest Conversion on Soil Bacterial Community Composition and Diversity in Subtropical Forests. CATENA.

[15] Chen Y., Martinez A., Cleavenger S., Rudolph J., Barberán A. (2021). Changes in Soil Microbial Communities across an Urbanization Gradient: A Local-Scale Temporal Study in the Arid Southwestern USA. Microorganisms.

[16] Dusengemungu L., Mubemba B., Gwanama C. (2022). Evaluation of Heavy Metal Contamination in Copper Mine Tailing Soils of Kitwe and Mufulira, Zambia, for Reclamation Prospects. Sci. Rep..

[17] Petänen T., Romantschuk M. (2003). Toxicity and Bioavailability to Bacteria of Particle-Associated Arsenite and Mercury. Chemosphere.

[18] Kang X., Yu X., Zhang Y., Cui Y., Tu W., Wang Q., Li Y., Hu L., Gu Y., Zhao K. (2018). Inoculation of Sinorhizobium Saheli YH1 Leads to Reduced Metal Uptake for *Leucaena leucocephala* Grown in Mine Tailings and Metal-Polluted Soils. Front. Microbiol..

[19] Jiwan S., Ajay K. (2011). Effects of Heavy Metals on Soil, Plants, Human Health and Aquatic Life. Int. J. Res. Chem. Environ..

[20] Azarbad H., Van Gestel C.A.M., Niklińska M., Laskowski R., Röling W.F.M., Van Straalen N.M. (2016). Resilience of Soil Microbial Communities to Metals and Additional Stressors: DNA-Based Approaches for Assessing “Stress-on-Stress” Responses. Int. J. Mol. Sci..

[21] Nkongolo K.K., Spiers G., Beckett P., Narendrula-Kotha R. (2022). Effects of Phytoremediation on Microbial Biomass, Composition, and Function in a Sulphide-Rich Tailing From a Metal-Contaminated Region. Front. Environ. Sci..

[22] Nkongolo K.K., Spiers G., Beckett P., Narendrula R., Theriault G., Tran A., Kalubi K.N. (2013). Long-Term Effects of Liming on Soil Chemistry in Stable and Eroded Upland Areas in a Mining Region. Water Air Soil Pollut..

[23] Nkongolo K.K., Michael P., Theriault G., Narendrula R., Castilloux P., Kalubi K.N., Beckett P., Spiers G. (2016). Assessing Biological Impacts of Land Reclamation in a Mining Region in Canada: Effects of Dolomitic Lime Applications on Forest Ecosystems and Microbial Phospholipid Fatty Acid Signatures. Water Air Soil Pollut..

[24] Abedin J., Beckett P., Spiers G. (2012). An Evaluation of Extractants for Assessment of Metal Phytoavailability to Guide Reclamation Practices in Acidic Soilscapes in Northern Regions. Can. J. Soil Sci..

[25] Schoch C.L., Seifert K., Huhndorf S., Robert V., Spouge J.L., Levesque C., Chen W., Consortium F.B., Bolchacova E., Voigt K. (2012). Nuclear Ribosomal Internal Transcribed Spacer (ITS) Region as a Universal DNA Barcode Marker for Fungi. Proc. Natl. Acad. Sci. USA.

[26] Edgar R.C., Bateman A. (2010). Search and Clustering Orders of Magnitude Faster than BLAST. Bioinformatics.

[27] Eren A.M., Zozaya M., Taylor C.M., Dowd S.E., Martin D.H., Ferris M.J. (2011). Exploring the Diversity of *Gardnerella Vaginalis* in the Genitourinary Tract Microbiota of Monogamous Couples Through Subtle Nucleotide Variation. PLoS ONE.

[28] Swanson K.S., Dowd S.E., Suchodolski J.S., Middelbos I.S., Vester B.M., Barry K.A., Nelson K.E., Torralba M., Henrissat B., Coutinho P.M. (2011). Phylogenetic and Gene-Centric Metagenomics of the Canine Intestinal Microbiome Reveals Similarities with Humans and Mice. ISME J..

[29] Bolyen E., Rideout J.R., Dillon M.R., Bokulich N.A., Abnet C.C., Al-Ghalith G.A., Alexander H., Alm E.J., Arumugam M., Asnicar F. (2019). Reproducible, Interactive, Scalable and Extensible Microbiome Data Science Using QIIME 2. Nat. Biotechnol..

[30] Dowd S.E., Callaway T.R., Wolcott R.D., Sun Y., McKeehan T., Hagevoort R.G., Edrington T.S. (2008). Evaluation of the Bacterial Diversity in the Feces of Cattle Using 16S RDNA Bacterial Tag-Encoded FLX Amplicon Pyrosequencing (BTEFAP). BMC Microbiol..

[31] Dowd S.E., Sun Y., Wolcott R.D., Domingo A., Carroll J.A. (2008). Bacterial Tag–Encoded FLX Amplicon Pyrosequencing (BTEFAP) for Microbiome Studies: Bacterial Diversity in the Ileum of Newly Weaned Salmonella-Infected Pigs. Foodborne Pathog. Dis..

[32] Karaca Y., Moonis M. (2022). Shannon Entropy-Based Complexity Quantification of Nonlinear Stochastic Process: Diagnostic and Predictive Spatiotemporal Uncertainty of Multiple Sclerosis Subgroups. Multi-Chaos, Fractal and Multi-Fractional Artificial Intelligence of Different Complex Systems.

[33] McCune B., Grace J.B., Urban D.L. (2002). Analysis of Ecological Communities.

[34] Pielou E.C. (1966). The Measurement of Diversity in Different Types of Biological Collections. J. Theor. Biol..

[35] Faith D.P. (1992). Conservation Evaluation and Phylogenetic Diversity. Biol. Conserv..

[36] Faith D.P., Scherson R.A., Faith D.P. (2018). Phylogenetic Diversity and Conservation Evaluation: Perspectives on Multiple Values, Indices, and Scales of Application. Phylogenetic Diversity: Applications and Challenges in Biodiversity Science.

[37] Islam K.R., Weil R.R. (2000). Soil Quality Indicator Properties in Mid-Atlantic Soils as Influenced by Conservation Management. J. Soil Water Conserv..

[38] Giertz S., Junge B., Diekkrüger B. (2005). Assessing the Effects of Land Use Change on Soil Physical Properties and Hydrological Processes in the Sub-Humid Tropical Environment of West Africa. Phys. Chem. Earth Parts A/B/C.

[39] de Quadros P.D., Zhalnina K., Davis-Richardson A.G., Drew J.C., Menezes F.B., Camargo F.A.d.O., Triplett E.W. (2016). Coal Mining Practices Reduce the Microbial Biomass, Richness and Diversity of Soil. Appl. Soil Ecol..

[40] Wu W., Dong C., Wu J., Liu X., Wu Y., Chen X., Yu S. (2017). Ecological Effects of Soil Properties and Metal Concentrations on the Composition and Diversity of Microbial Communities Associated with Land Use Patterns in an Electronic Waste Recycling Region. Sci. Total Environ..

[41] Zeng D.H., Hu Y.L., Chang S.X., Fan Z.P. (2009). Land Cover Change Effects on Soil Chemical and Biological Properties after Planting Mongolian Pine (Pinus Sylvestris Var. Mongolica) in Sandy Lands in Keerqin, Northeastern China. Plant Soil.

[42] Moghimian N., Hosseini S.M., Kooch Y., Darki B.Z. (2017). Impacts of Changes in Land Use/Cover on Soil Microbial and Enzyme Activities. CATENA.

[43] Hijbeek R., Cormont A., Hazeu G., Bechini L., Zavattaro L., Janssen B., Werner M., Schlatter N., Guzmán G., Bijttebier J. (2017). Do Farmers Perceive a Deficiency of Soil Organic Matter? A European and Farm Level Analysis. Ecol. Indic..

[44] Bradl H.B. (2004). Adsorption of Heavy Metal Ions on Soils and Soils Constituents. J. Colloid Interface Sci..

[45] Painter S., Cameron E.M., Allan R., Rouse J. (1994). Reconnaissance Geochemistry and Its Environmental Relevance. J. Geochem. Explor..

[46] Narendrula R., Nkongolo K.K., Beckett P. (2012). Comparative Soil Metal Analyses in Sudbury (Ontario, Canada) and Lubumbashi (Katanga, DR-Congo). Bull. Environ. Contam. Toxicol..

[47] Tembo B.D., Sichilongo K., Cernak J. (2006). Distribution of Copper, Lead, Cadmium and Zinc Concentrations in Soils around Kabwe Town in Zambia. Chemosphere.

[48] Mehes-Smith M., Nkongolo K.K. (2015). Physiological and Cytological Responses of Deschampsia Cespitosa and Populus Tremuloides to Soil Metal Contamination. Water. Air. Soil Pollut..

[49] Mehes-Smith M., Nkongolo K., Cholew E., Silvern S., Young S. (2013). Coping Mechanisms of Plants to Metal Contaminated Soil. Environmental Change and Sustainability.

[50] Foy C.D., Chaney R.L., White M.C. (1978). The Physiology of Metal Toxicity in Plants. Annu. Rev. Plant Physiol..

[51] Benavides M., Gallego S., Tomaro M. (2005). Cadmium Toxicity in Plants. Plant Physiol..

[52] Mununga Katebe F., Raulier P., Colinet G., Ngoy Shutcha M., Mpundu Mubemba M., Jijakli M.H. (2023). Assessment of Heavy Metal Pollution of Agricultural Soil, Irrigation Water, and Vegetables in and Nearby the Cupriferous City of Lubumbashi, (Democratic Republic of the Congo). Agronomy.

[53] Malaisse F., Gregoire J. (1978). Contribution à la phytogéochimie de la mine de l’étoile (shaba, zaïre). Bull. Société R. Bot. Belg./Bull. Van K. Belg. Bot. Ver..

[54] Yang T.-T., Liu J., Chen W.C., Chen X., Shu H.Y., Jia P., Liao B., Shu W.S., Li J.T. (2017). Changes in Microbial Community Composition Following Phytostabilization of an Extremely Acidic Cu Mine Tailings. Soil Biol. Biochem..

[55] Li Y., Jia Z., Sun Q., Zhan J., Yang Y., Wang D. (2016). Ecological Restoration Alters Microbial Communities in Mine Tailings Profiles. Sci. Rep..

[56] Singh B.K., Quince C., Macdonald C.A., Khachane A., Thomas N., Al-Soud W.A., Sørensen S.J., He Z., White D., Sinclair A. (2014). Loss of Microbial Diversity in Soils Is Coincident with Reductions in Some Specialized Functions. Environ. Microbiol..

[57] Chen J., He F., Zhang X., Sun X., Zheng J., Zheng J. (2014). Heavy Metal Pollution Decreases Microbial Abundance, Diversity and Activity within Particle-Size Fractions of a Paddy Soil. FEMS Microbiol. Ecol..

[58] Chodak M., Gołebiewski M., Morawska-Płoskonka J., Kuduk K., Niklińska M. (2013). Diversity of Microorganisms from Forest Soils Differently Polluted with Heavy Metals. Appl. Soil Ecol..

[59] Hong C., Si Y., Xing Y., Li Y. (2015). Illumina MiSeq Sequencing Investigation on the Contrasting Soil Bacterial Community Structures in Different Iron Mining Areas. Environ. Sci. Pollut. Res..

[60] Berg J., Brandt K.K., Al-Soud W.A., Holm P.E., Hansen L.H., Sørensen S.J., Nybroe O. (2012). Selection for Cu-Tolerant Bacterial Communities with Altered Composition, but Unaltered Richness, via Long-Term Cu Exposure. Appl. Environ. Microbiol..

[61] Nkongolo K.K., Narendrula-Kotha R. (2020). Advances in Monitoring Soil Microbial Community Dynamic and Function. J. Appl. Genet..

[62] Brandt K.K., Frandsen R.J.N., Holm P.E., Nybroe O. (2010). Development of Pollution-Induced Community Tolerance Is Linked to Structural and Functional Resilience of a Soil Bacterial Community Following a Five-Year Field Exposure to Copper. Soil Biol. Biochem..

[63] Deng S., Ke T., Li L., Cai S., Zhou Y., Liu Y., Guo L., Chen L., Zhang D. (2018). Impacts of Environmental Factors on the Whole Microbial Communities in the Rhizosphere of a Metal-Tolerant Plant: Elsholtzia Haichowensis Sun. Environ. Pollut..

[64] Kuang J.L., Huang L.N., Chen L.X., Hua Z.S., Li S.J., Hu M., Li J.T., Shu W.S. (2012). Contemporary Environmental Variation Determines Microbial Diversity Patterns in Acid Mine Drainage. ISME J..

[65] Lozupone C., Lladser M.E., Knights D., Stombaugh J., Knight R. (2010). UniFrac: An Effective Distance Metric for Microbial Community Comparison. ISME J..

[66] Lozupone C.A., Hamady M., Kelley S.T., Knight R. (2007). Quantitative and Qualitative β Diversity Measures Lead to Different Insights into Factors That Structure Microbial Communities. Appl. Environ. Microbiol..

[67] Zhao H., Xia B., Fan C., Zhao P., Shen S. (2012). Human Health Risk from Soil Heavy Metal Contamination under Different Land Uses near Dabaoshan Mine, Southern China. Sci. Total Environ..

[68] Li J., Zheng Y.M., Liu Y.R., Ma Y.B., Hu H.W., He J.Z. (2014). Initial Copper Stress Strengthens the Resistance of Soil Microorganisms to a Subsequent Copper Stress. Microb. Ecol..

[69] Bourceret A., Cébron A., Tisserant E., Poupin P., Bauda P., Beguiristain T., Leyval C. (2016). The Bacterial and Fungal Diversity of an Aged PAH- and Heavy Metal-Contaminated Soil Is Affected by Plant Cover and Edaphic Parameters. Microb. Ecol..

[70] Gołebiewski M., Deja-Sikora E., Cichosz M., Tretyn A., Wróbel B. (2014). 16S RDNA Pyrosequencing Analysis of Bacterial Community in Heavy Metals Polluted Soils. Microb. Ecol..

[71] Li J., Hu H.W., Ma Y.B., Wang J.T., Liu Y.R., He J.Z. (2015). Long-Term Nickel Exposure Altered the Bacterial Community Composition but Not Diversity in Two Contrasting Agricultural Soils. Environ. Sci. Pollut. Res..

[72] Bååth E., Frostegård Å., Díaz-Raviña M., Tunlid A. (1998). Microbial Community-Based Measurements to Estimate Heavy Metal Effects in Soil: The Use of Phospholipid Fatty Acid Patterns and Bacterial Community Tolerance. Ambio.

[73] Bååth E., Díaz-Raviña M., Frostegård Å., Campbell C.D. (1998). Effect of Metal-Rich Sludge Amendments on the Soil Microbial Community. Appl. Environ. Microbiol..

[74] Pennanen T., Frostegård Å., Fritze H., Bååth E. (1996). Phospholipid Fatty Acid Composition and Heavy Metal Tolerance of Soil Microbial Communities along Two Heavy Metal-Polluted Gradients in Coniferous Forests. Appl. Environ. Microbiol..

[75] Kelly J.J., Häggblom M., Tate R.L., Kelly J.J., Häggblom M., Tate R.L. (1999). Effects of the Land Application of Sewage Sludge on Soil Heavy Metal Concentrations and Soil Microbial Communities. SBiBi.

[76] Witter E., Gong P., Bååth E., Marstorp H. (2000). A Study of the Structure and Metal Tolerance of the Soil Microbial Community Six Years after Cessation of Sewage Sludge Applications. Environ. Toxicol. Chem..

[77] Harris-Hellal J., Vallaeys T., Garnier-Zarli E., Bousserrhine N. (2009). Effects of Mercury on Soil Microbial Communities in Tropical Soils of French Guyana. Appl. Soil Ecol..

[78] Oburger E., Schmidt H. (2016). New Methods To Unravel Rhizosphere Processes. Trends Plant Sci..

[79] Mendes R., Garbeva P., Raaijmakers J.M. (2013). The Rhizosphere Microbiome: Significance of Plant Beneficial, Plant Pathogenic, and Human Pathogenic Microorganisms. FEMS Microbiol. Rev..

[80] Yan X., An J., Yin Y., Gao C., Wang B., Wei S. (2022). Heavy Metals Uptake and Translocation of Typical Wetland Plants and Their Ecological Effects on the Coastal Soil of a Contaminated Bay in Northeast China. Sci. Total Environ..

[81] Li C., Quan Q., Gan Y., Dong J., Fang J., Wang L., Liu J. (2020). Effects of Heavy Metals on Microbial Communities in Sediments and Establishment of Bioindicators Based on Microbial Taxa and Function for Environmental Monitoring and Management. Sci. Total Environ..

[82] Xiao Y., Chen L., Li C., Ma J., Chen R., Yang B., Liu G., Liu S., Fang J. (2023). Role of the Rhizosphere Bacterial Community in Assisting Phytoremediation in a Lead-Zinc Area. Front. Plant Sci..

[83] Zhao X., Huang J., Zhu X., Chai J., Ji X. (2020). Ecological Effects of Heavy Metal Pollution on Soil Microbial Community Structure and Diversity on Both Sides of a River around a Mining Area. Int. J. Environ. Res. Public Health.

