# Supplementary material for: Soil Microbial Responses to Varying Environmental Conditions in a Copper Belt Region of Africa: Phytoremediation Perspectives
Source: Microorganisms. 2024 Dec 27;13(1):31. doi: 10.3390/microorganisms13010031 (PMC11767397; doi:10.3390/microorganisms13010031)
Supplement: Supplementary file 1 [file microorganisms-13-00031-s001.zip › microorganisms-3371119-supplementary.pdf]

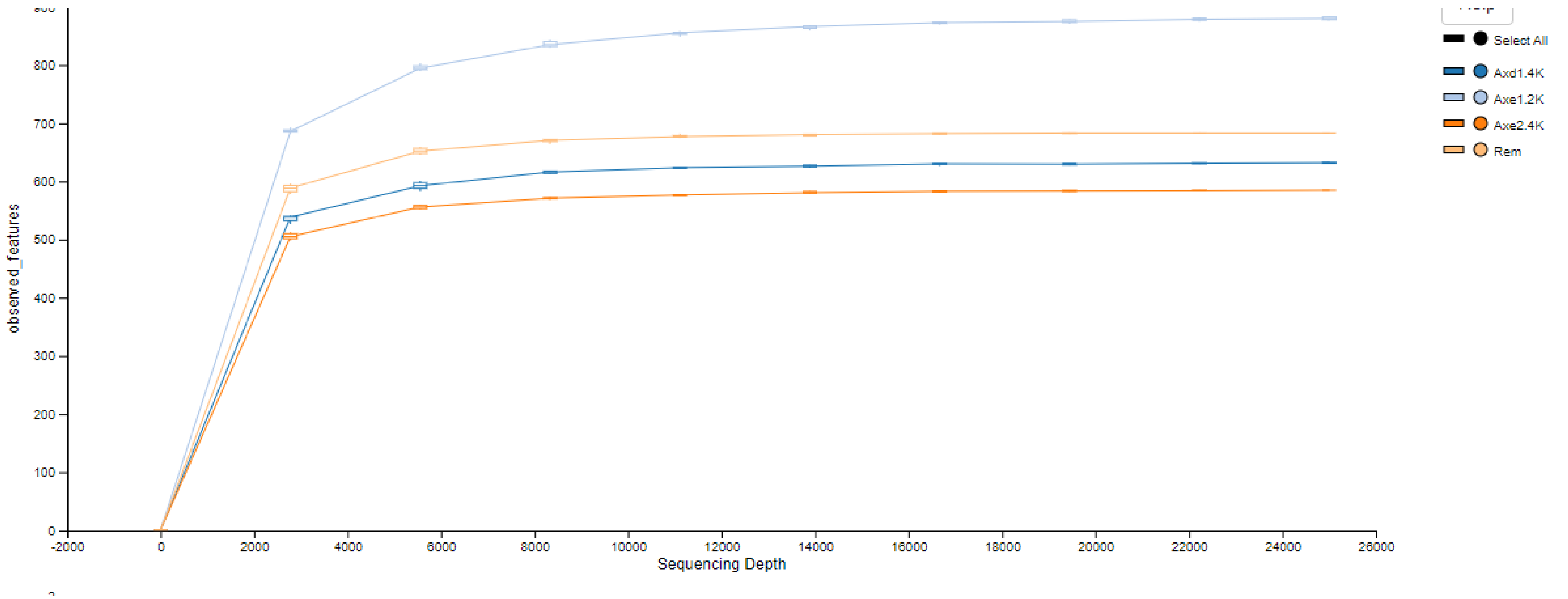

Figure S1. Observed Features (amplicon Sequence Variants) chart for bacterial communities. It shows that the plateau for each sample was achieved. The most observed features were found in sample from Axe-1-2K (878 - ASV) and the least in samples from Axe2-4K (585 features - ASV). Axe1-2K: residential site; Axe1-4K or Axd1-4K: agricultural dry land, Axe2-4K: agricultural wet land, and REM: Remediated tailing.

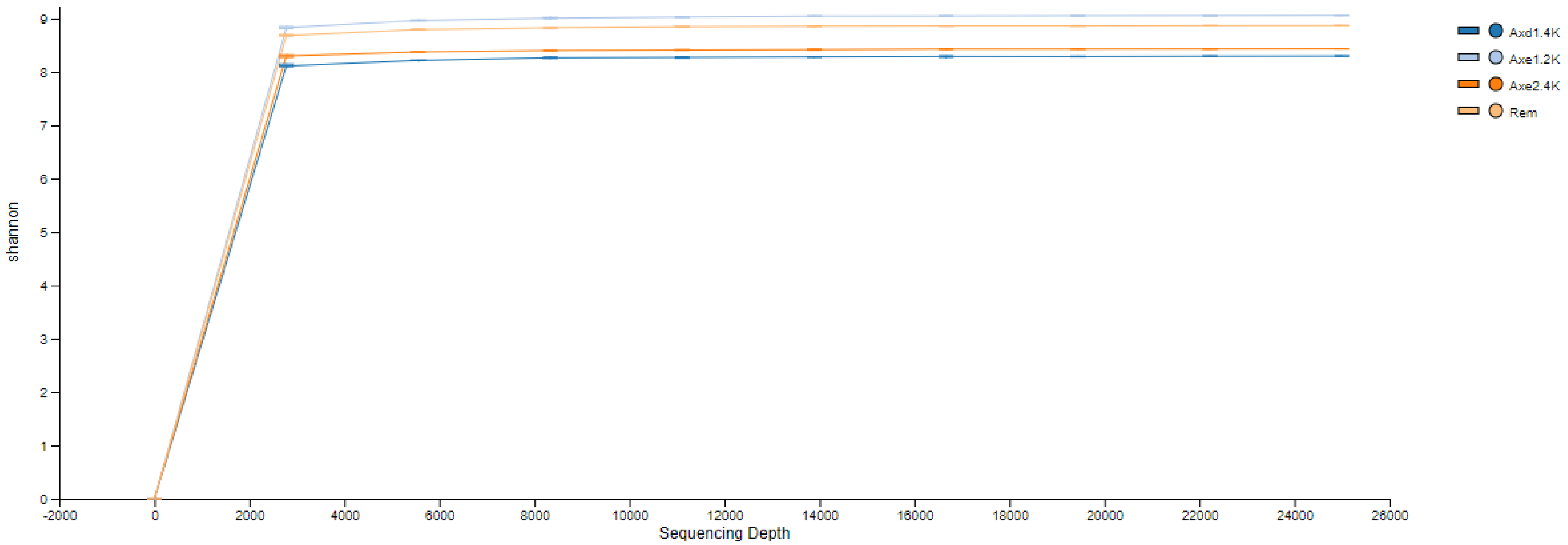

Figure S2. Shannon Diversity Entropy for bacterial communities showing the levels of diversity within each site based on Amplicon Sequences Variants. Axe1-2K: residential site; Axe1-4K or Axd1-4K: agricultural dry land, Axe2-4K: agricultural wet land, and REM: Remediated tailing.

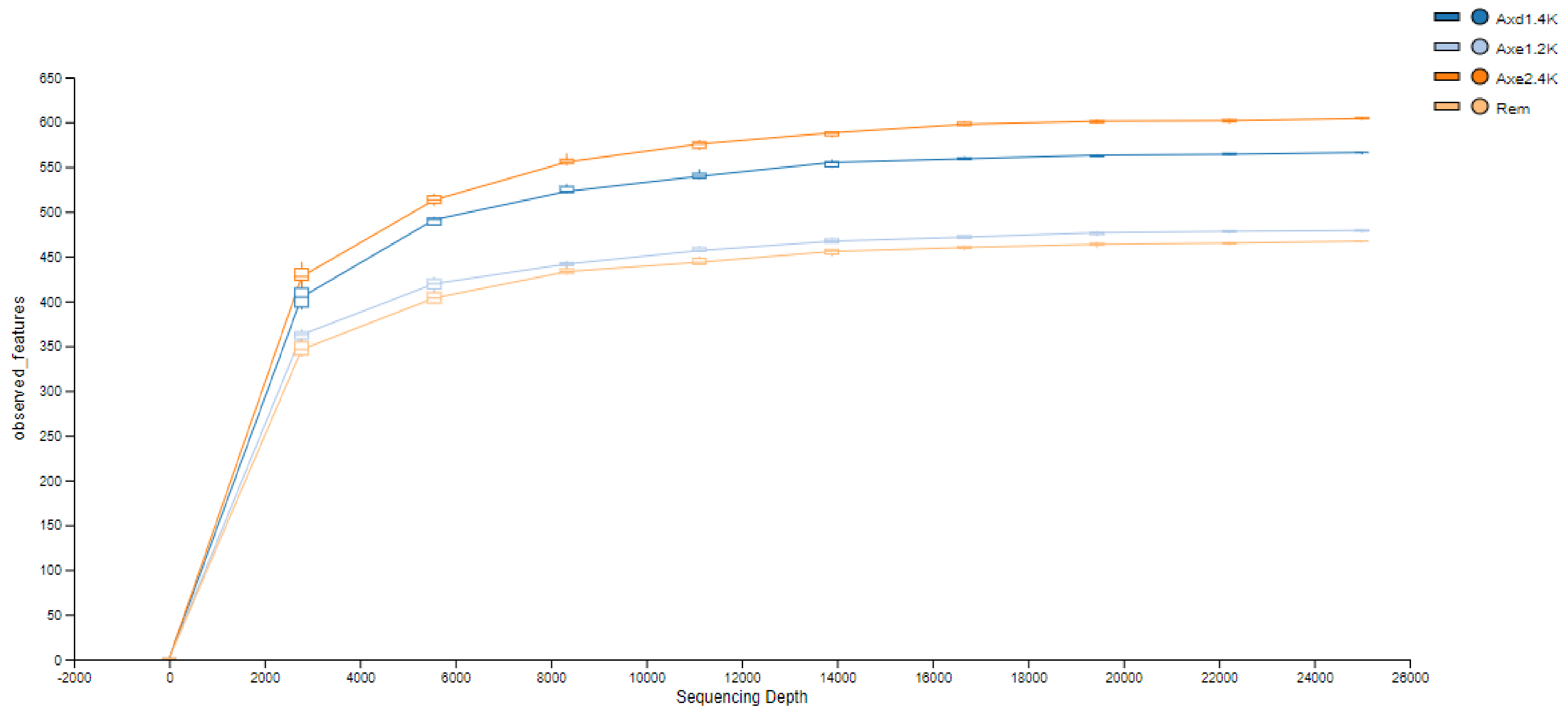

Figure S3. Observed Features chart for fungal communities that Illustrate that the plateau was achieved for each sample. The most observed features were found in sample Axe1-2K (878) (Axe4-605) and the least in sample Axe2-4K (585 features) (Axe1-2K (479). Axe1-2K: residential site; Axe1-4K or Axd1-4K: agricultural dry land, Axe2-4k: agricultural wet land, and REM: Remediated tailing.

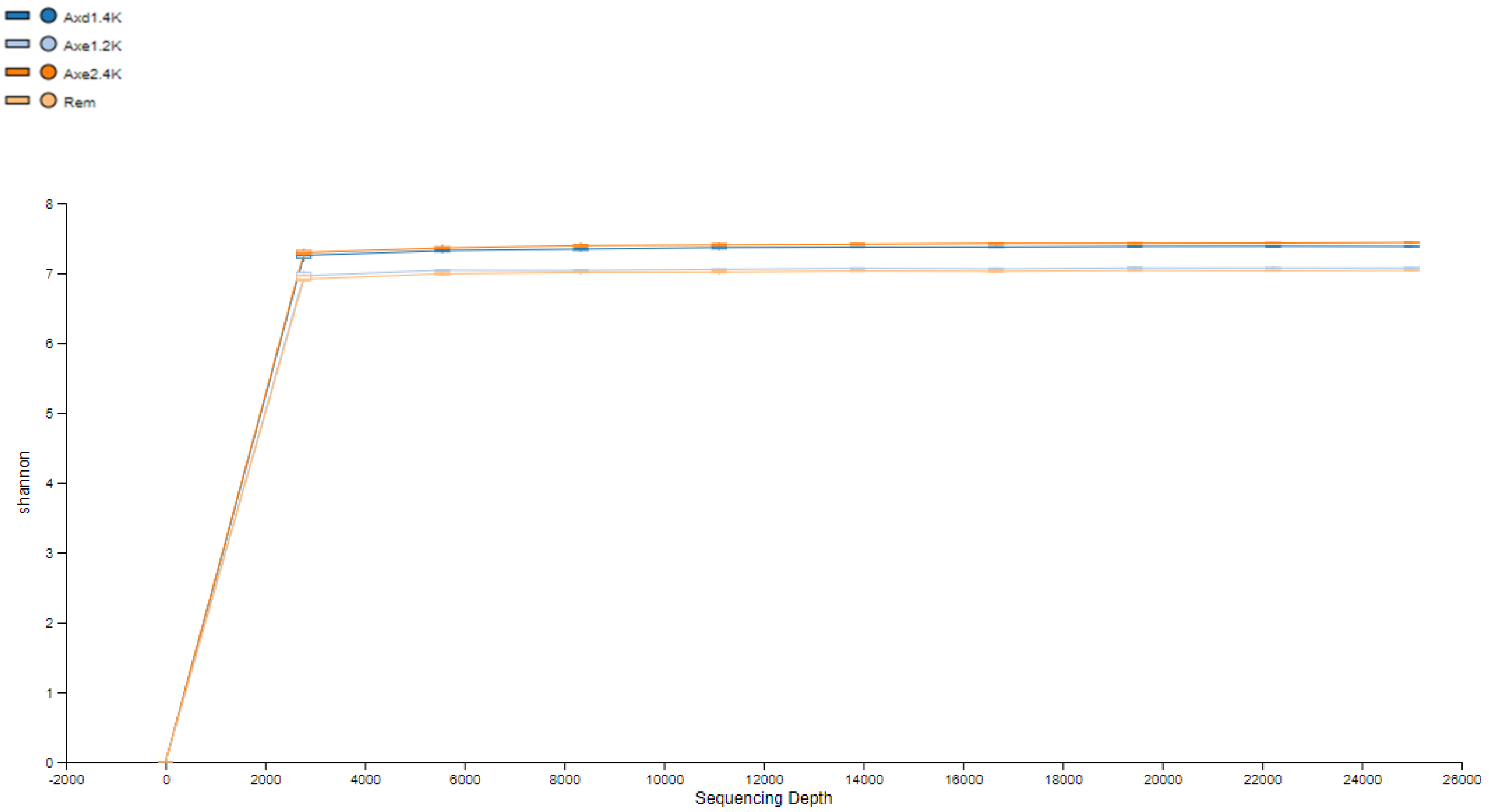

Figure S4: Shannon Diversity Entropy for fungal communities showing the levels of diversity within each Site. Axe1-2K: residential site; Axe1-4K or Axd1-4K: agricultural dry land, Axe2-4k: agricultural wet land, and REM: Remediated tailing.

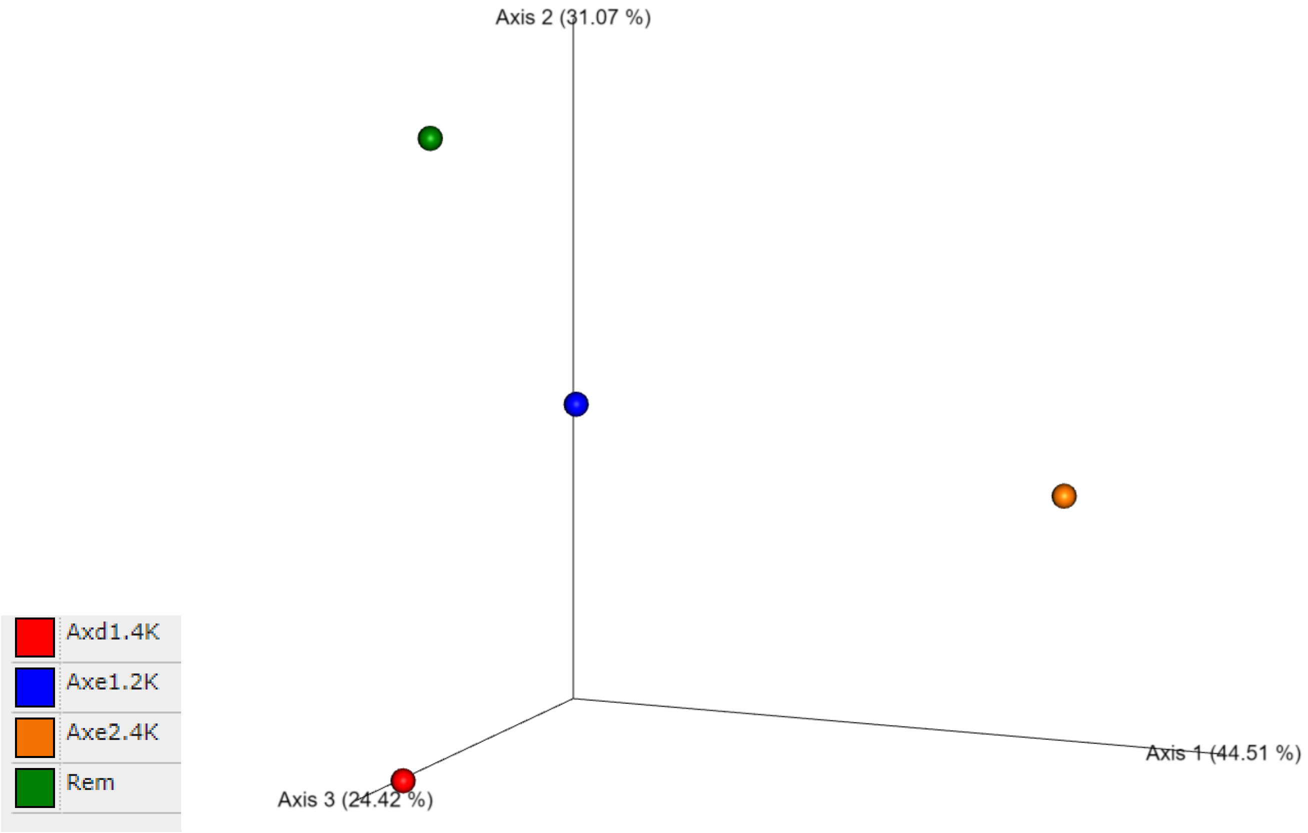

Figure S5. Principal coordinate plot of weighted UniFrac data for fungal communities
Colors keyed on the treatment group. Primary vector explains 73% of the variation between the groups. The first three vectors together exhibit over 88% of the variation among the groups.

Clear grouping of the samples can be observed (blue and green closest). a) Axe1-2K: site 1 (residential area); b) Axe1-4K or Axed1-4K: site 2 (agricultural dry land); c) Axe2-4K: site 3 (agricultural wet field); and d) Rem: Remediated tailing (site 4).

Table S1: Number of phyla, classes, families, and generation across targeted sites

Identity	Axis 1- 2K	Axis 1 – 4K	Axis – 4K	Remediation Tailing
Bacteria				
Phylum	16	13	15	17
Class	42	38	43	45
Family	181	168	190	188
Genus	351	326	389	375
Fungi				
Phylum	8	8	8	8
Class	30	30	27	31
Family	225	219	189	225
Genus	427	422	335	424

Axis 1-2K: residential site; Axis 2-4K: agricultural dry land, Axis 2 – 4K: agricultural wetland, and REM: Remediated tailing

Table S2: Bacteria Bray Curtis distance matrix

Sites	Site 2	Site 1	Site 3	Tailing (Site 4)
Site 2	-	0.93672	0.79016	0.90392
Site 1	0.93672	0	0.86248	0.8128
Site 3	0.79016	0.86248	0	0.82976
Tailing (site 4)	0.90392	0.8128	0.82976	0

Site 1: residential land; Site: represents agricultural dry land; Site 3: agricultural wetland, and site 4: Remediated tailing

Table S3. Bacteria Unweighted UniFrac distance matrix

	Site 2	Site 1	Site 3	Tailing (Site 4)
Site 2	0	0.831019	0.753383	0.817446
Site 1	0.831019	0	0.741709	0.72539
Site 3	0.753383	0.741709	0	0.760644
Tailing (Site 4)	0.817446	0.72539	0.760644	0

Site 1: residential land; Site 2: agricultural dry land; Site 3: agricultural wetland, and site 4: Remediated tailing

Table S4. Fungi - Bray Curtis distance matrix

	Site 3	Site 2	Site 1	Tailing (Site 4)
Site 3	0	0.98472	0.98452	0.98484
Site 2	0.98472	0	0.76444	0.76252
Site 1	0.98452	0.76444	0	0.778
Tailing (Site 4)	0.98484	0.76252	0.778	0

Site 1: residential land; Site 2: agricultural dry land; Site 3: agricultural wetland, and site 4: remediated tailing.

Table 5S. Fungi - Unweighted UniFrac distance matrix

	Site 3	Site 2	Site 1	Tailing (Site 4)
Site 3	0	0.665571	0.671074	0.716856
Site 2	0.665571	0	0.65643	0.681479
Site 1	0.671074	0.65643	0	0.616049
Tailing (Site 4)	0.716856	0.681479	0.616049	0

Site 1: residential land; Site 2: agricultural dry land; Site 3: agricultural wetland, and site 4: Remediated tailing

Table S6. Bacterial genera count and relative abundance across sampling sites

Genus	AXIS 1-2K		AXIS 1-4K		AXIS 2-4K		Remediation Site	
	Count	Relative Abundance	Count	Relative Abundance	Count	Relative Abundance	Count	Relative Abundance
paenibacillus	34	0.060	446	0.730	132	0.220	8	0.015
methylovorus	5	0.009	0	0.000	22	0.037	0	0.000
algisphaera	103	0.183	132	0.216	138	0.230	369	0.700
shewanella	0	0.000	0	0.000	0	0.000	22	0.042
amphritea	249	0.442	20	0.033	93	0.155	52	0.099
thermodesulfovibrio	27	0.048	3	0.005	15	0.025	120	0.228
planctomyces	169	0.300	47	0.077	84	0.140	124	0.235
glaciimonas	0	0.000	70	0.115	4	0.007	17	0.032
acidithrix	0	0.000	26	0.043	5	0.008	17	0.032
chondromyces	153	0.271	85	0.139	134	0.223	214	0.406
actinoplanes	7	0.012	22	0.036	20	0.033	23	0.044
caldalkalibacillus	0	0.000	118	0.193	0	0.000	0	0.000
ectothiorhodospira	121	0.215	0	0.000	33	0.055	48	0.091
dyadobacter	26	0.046	1	0.002	1	0.002	14	0.027
nitrosomonas	18	0.032	6	0.010	18	0.030	117	0.222
nonomuraea	16	0.028	4	0.007	6	0.010	52	0.099
fibrobacter	1	0.002	0	0.000	0	0.000	30	0.057
thermolithobacter	36	0.064	49	0.080	19	0.032	11	0.021
anaeromyxobacter	198	0.351	154	0.252	325	0.542	115	0.218
ensifer	13	0.023	8	0.013	85	0.142	4	0.008
candidatus entotheonella	2	0.004	1	0.002	4	0.007	135	0.256
acidothermus	55	0.098	53	0.087	100	0.167	41	0.078
ferritrophicum	0	0.000	14	0.023	0	0.000	0	0.000
candidatus solibacter	854	1.515	2425	3.968	1658	2.763	1074	2.038
candidatus anammoximicrobium	47	0.083	54	0.088	106	0.177	92	0.175
agromyces	713	1.265	4	0.007	121	0.202	115	0.218
zoogloea	0	0.000	0	0.000	150	0.250	1	0.002
microbacterium	15	0.027	0	0.000	5	0.008	6	0.011
rhodovibrio	21	0.037	29	0.047	13	0.022	21	0.040
vallitalea	0	0.000	27	0.044	2	0.003	10	0.019
marmoricola	16	0.028	17	0.028	42	0.070	18	0.034
gemmatimonas	1738	3.083	671	1.098	1813	3.022	1541	2.925
segetibacter	0	0.000	0	0.000	0	0.000	12	0.023
heliophilum	0	0.000	49	0.080	2	0.003	0	0.000
blastopirellula	123	0.218	25	0.041	46	0.077	96	0.182
spongiibacter	28	0.050	0	0.000	8	0.013	2	0.004
nakamurella	8	0.014	5	0.008	35	0.058	18	0.034
rhodobium	1	0.002	1	0.002	69	0.115	10	0.019
hydrocarboniphaga	281	0.498	1	0.002	5	0.008	15	0.028
sinorhizobium	13	0.023	10	0.016	27	0.045	24	0.046
azoarcus	232	0.412	27	0.044	154	0.257	353	0.670

Table S6. Bacterial genera count and relative abundance across sampling sites

Genus	AXIS 1-2K		AXIS 1-4K		AXIS 2-4K		Remediation Site	
	Count	Relative Abundance	Count	Relative Abundance	Count	Relative Abundance	Count	Relative Abundance
gracilibacter	23	0.041	1	0.002	3	0.005	14	0.027
desulfobacca	206	0.365	6	0.010	28	0.047	22	0.042
melghirimyces	86	0.153	51	0.083	53	0.088	84	0.159
georgenia	391	0.694	36	0.059	29	0.048	4	0.008
kaistobacter	331	0.587	8	0.013	244	0.407	54	0.102
pseudomonas	36	0.064	8	0.013	126	0.210	63	0.120
cryptosporangium	3	0.005	1	0.002	8	0.013	8	0.015
salinispora	0	0.000	32	0.052	2	0.003	1	0.002
pseudenhygromyxa	9	0.016	0	0.000	1	0.002	1	0.002
nordella	67	0.119	38	0.062	190	0.317	130	0.247
georgfuchsia	6	0.011	2	0.003	32	0.053	5	0.009
streptacidiphilus	8	0.014	6	0.010	350	0.583	2	0.004
niastella	121	0.215	13	0.021	46	0.077	108	0.205
desertibacter	0	0.000	25	0.041	11	0.018	20	0.038
jahnella	20	0.035	6	0.010	27	0.045	31	0.059
mizugakiibacter	1	0.002	0	0.000	301	0.502	41	0.078
catellatospora	5	0.009	48	0.079	46	0.077	12	0.023
hydrogenispora	412	0.731	19	0.031	29	0.048	7	0.013
azospirillum	91	0.161	243	0.398	192	0.320	20	0.038
oryzihumus	0	0.000	2	0.003	156	0.260	0	0.000
haliea	15	0.027	0	0.000	67	0.112	36	0.068
ethanoligenens	0	0.000	19	0.031	11	0.018	0	0.000
microbispora	1	0.002	29	0.047	5	0.008	0	0.000
pyrinomonas	154	0.273	10	0.016	7	0.012	510	0.968
diplosphaera	12	0.021	1	0.002	5	0.008	27	0.051
anoxybacillus	19	0.034	329	0.538	46	0.077	3	0.006
lentzea	82	0.145	5	0.008	8	0.013	10	0.019
candidatus jettenia	9	0.016	0	0.000	1	0.002	9	0.017
singulisphaera	13	0.023	317	0.519	181	0.302	78	0.148
microvirga	261	0.463	440	0.720	732	1.220	536	1.017
labrys	4	0.007	30	0.049	49	0.082	9	0.017
dongia	294	0.521	16	0.026	158	0.263	432	0.820
caldicoprobacter	0	0.000	27	0.044	2	0.003	0	0.000
lysinibacillus	20	0.035	71	0.116	17	0.028	0	0.000
labrenzia	2	0.004	0	0.000	21	0.035	33	0.063
lautropia	12	0.021	1	0.002	4	0.007	15	0.028
rhizobacter	10	0.018	0	0.000	16	0.027	18	0.034
chitinophaga	185	0.328	17	0.028	72	0.120	115	0.218
ruminococcus	9	0.016	5	0.008	12	0.020	29	0.055
actinomadura	8	0.014	964	1.577	343	0.572	82	0.156
thermotoga	0	0.000	0	0.000	0	0.000	28	0.053

Table S6. Bacterial genera count and relative abundance across sampling sites

Genus	AXIS 1-2K		AXIS 1-4K		AXIS 2-4K		Remediation Site	
	Count	Relative Abundance	Count	Relative Abundance	Count	Relative Abundance	Count	Relative Abundance
pseudoduganella	12	0.021	1	0.002	2	0.003	69	0.131
collimonas	103	0.183	18	0.029	28	0.047	15	0.028
lewinella	44	0.078	0	0.000	19	0.032	33	0.063
roseomonas	150	0.266	122	0.200	50	0.083	132	0.251
devosia	197	0.349	31	0.051	381	0.635	222	0.421
kitasatospora	0	0.000	0	0.000	2	0.003	20	0.038
magnetospira	10	0.018	0	0.000	7	0.012	2	0.004
pseudonocardia	209	0.371	177	0.290	130	0.217	505	0.958
granulicella	0	0.000	86	0.141	84	0.140	75	0.142
oceanobacillus	0	0.000	0	0.000	0	0.000	18	0.034
caloramator	0	0.000	24	0.039	104	0.173	33	0.063
cupriavidus	4	0.007	22	0.036	38	0.063	13	0.025
alicycliphilus	21	0.037	2	0.003	19	0.032	28	0.053
acidisphaera	0	0.000	4	0.007	11	0.018	7	0.013
haliangium	318	0.564	211	0.345	251	0.418	361	0.685
phycicoccus	107	0.190	131	0.214	673	1.122	19	0.036
tessaracoccus	0	0.000	31	0.051	12	0.020	7	0.013
castellaniella	0	0.000	0	0.000	96	0.160	0	0.000
planifilum	0	0.000	73	0.119	0	0.000	0	0.000
afifella	188	0.333	0	0.000	6	0.010	159	0.302
hymenobacter	1	0.002	0	0.000	0	0.000	30	0.057
actinomycetospora	13	0.023	131	0.214	18	0.030	48	0.091
ktedonobacter	0	0.000	1660	2.716	349	0.582	283	0.537
methyloligella	20	0.035	16	0.026	99	0.165	75	0.142
frateuria	4	0.007	8	0.013	101	0.168	21	0.040
thermoleophilum	366	0.649	1841	3.013	395	0.658	937	1.778
salinarimonas	1	0.002	2	0.003	1	0.002	56	0.106
paracraurococcus	3	0.005	8	0.013	10	0.017	7	0.013
ornithinimicrobium	66	0.117	2	0.003	4	0.007	0	0.000
oxalophagus	4	0.007	295	0.483	53	0.088	0	0.000
desulfosalsimonas	30	0.053	1	0.002	0	0.000	29	0.055
actinospica	0	0.000	79	0.129	8	0.013	1	0.002
blastochloris	99	0.176	208	0.340	103	0.172	262	0.497
geodermatophilus	218	0.387	558	0.913	121	0.202	404	0.767
massilia	203	0.360	165	0.270	363	0.605	205	0.389
thermosipho	0	0.000	0	0.000	0	0.000	28	0.053
variovorax	7	0.012	18	0.029	37	0.062	65	0.123
blastocatella	112	0.199	0	0.000	2	0.003	27	0.051
lachnoclostridium	54	0.096	60	0.098	30	0.050	8	0.015
staphylococcus	6	0.011	7	0.011	0	0.000	16	0.030
corallococcus	35	0.062	16	0.026	48	0.080	14	0.027

Table S6. Bacterial genera count and relative abundance across sampling sites

Genus	AXIS 1-2K		AXIS 1-4K		AXIS 2-4K		Remediation Site	
	Count	Relative Abundance	Count	Relative Abundance	Count	Relative Abundance	Count	Relative Abundance
rhodanobacter	5	0.009	0	0.000	626	1.043	5	0.009
actinotalea	113	0.200	3	0.005	38	0.063	36	0.068
ferrimicrobium	7	0.012	1	0.002	6	0.010	8	0.015
ohtaekwangia	828	1.469	0	0.000	16	0.027	272	0.516
rugosimonospora	0	0.000	27	0.044	17	0.028	3	0.006
anaerolinea	31	0.055	0	0.000	1	0.002	20	0.038
lechevalieria	96	0.170	0	0.000	3	0.005	118	0.224
pirellula	195	0.346	15	0.025	72	0.120	126	0.239
alkalilimnicola	0	0.000	27	0.044	12	0.020	10	0.019
thermodesulforhabdus	24	0.043	7	0.011	44	0.073	103	0.195
novosphingobium	126	0.223	19	0.031	287	0.478	39	0.074
moorella	17	0.030	0	0.000	0	0.000	80	0.152
nitrobacter	2	0.004	2	0.003	121	0.202	9	0.017
candidatus atelocyanobacterium	262	0.465	80	0.131	94	0.157	191	0.362
denitratisoma	0	0.000	210	0.344	66	0.110	7	0.013
gaiella	857	1.520	3793	6.207	1735	2.892	1007	1.911
arthrobacter	476	0.844	428	0.700	3055	5.091	287	0.545
burkholderia	307	0.545	262	0.429	374	0.623	261	0.495
gemmobacter	31	0.055	0	0.000	4	0.007	24	0.046
ochrobactrum	105	0.186	9	0.015	32	0.053	30	0.057
prolixibacter	5	0.009	0	0.000	7	0.012	5	0.009
desulfomonile	12	0.021	34	0.056	20	0.033	5	0.009
diaminobutyricimonas	17	0.030	37	0.061	33	0.055	137	0.260
hyphomicrobium	241	0.427	18	0.029	699	1.165	342	0.649
microcoleus	58	0.103	593	0.970	0	0.000	77	0.146
acetomicrobium	0	0.000	111	0.182	0	0.000	0	0.000
coprococcus	45	0.080	0	0.000	7	0.012	2	0.004
aquincola	99	0.176	36	0.059	60	0.100	47	0.089
brevundimonas	13	0.023	7	0.011	50	0.083	10	0.019
litorilinea	72	0.128	11	0.018	54	0.090	2	0.004
cycloclasticus	84	0.149	1	0.002	6	0.010	78	0.148
solibacillus	12	0.021	24	0.039	22	0.037	1	0.002
rubrobacter	107	0.190	69	0.113	49	0.082	162	0.307
desulfococcus	5	0.009	0	0.000	14	0.023	25	0.047
yonghaparkia	5	0.009	71	0.116	13	0.022	17	0.032
rhodoplanes	341	0.605	1022	1.672	1427	2.378	1279	2.427
desulfonema	7	0.012	35	0.057	50	0.083	71	0.135
bradyrhizobium	532	0.944	1093	1.789	1798	2.997	2140	4.061
vasilyevaea	12	0.021	0	0.000	11	0.018	23	0.044
sphingomonas	1880	3.335	1064	1.741	2578	4.297	766	1.454
cohnella	3	0.005	150	0.245	58	0.097	0	0.000

Table S6. Bacterial genera count and relative abundance across sampling sites

Genus	AXIS 1-2K		AXIS 1-4K		AXIS 2-4K		Remediation Site	
	Count	Relative Abundance	Count	Relative Abundance	Count	Relative Abundance	Count	Relative Abundance
actinoallomurus	2	0.004	934	1.528	327	0.545	27	0.051
salinibacterium	1	0.002	0	0.000	23	0.038	9	0.017
salinicoccus	2	0.004	0	0.000	10	0.017	11	0.021
pedomicrobium	58	0.103	3	0.005	98	0.163	132	0.251
ureibacillus	1013	1.797	445	0.728	659	1.098	844	1.602
desulfovibrio	31	0.055	10	0.016	10	0.017	25	0.047
porphyrobacter	57	0.101	5	0.008	102	0.170	17	0.032
aquabacterium	35	0.062	3	0.005	27	0.045	184	0.349
nitrospira	318	0.564	175	0.286	627	1.045	278	0.528
xenophilus	21	0.037	17	0.028	66	0.110	93	0.177
luteimonas	1	0.002	0	0.000	157	0.262	1	0.002
caldibacillus	30	0.053	5	0.008	8	0.013	10	0.019
shinella	120	0.213	2	0.003	40	0.067	64	0.121
lysobacter	107	0.190	6	0.010	441	0.735	34	0.065
caldanaerobacter	23	0.041	0	0.000	1	0.002	6	0.011
planococcus	0	0.000	39	0.064	2	0.003	1	0.002
herminiimonas	12	0.021	17	0.028	112	0.187	8	0.015
opitutus	351	0.623	19	0.031	26	0.043	168	0.319
flavitalea	149	0.264	4	0.007	45	0.075	100	0.190
sulfuritalea	50	0.089	3	0.005	14	0.023	9	0.017
micromonospora	52	0.092	119	0.195	146	0.243	87	0.165
candidatus chloroploca	39	0.069	0	0.000	12	0.020	2	0.004
brochothrix	11	0.020	12	0.020	8	0.013	11	0.021
phormidium	2	0.004	33	0.054	0	0.000	0	0.000
simkania	9	0.016	0	0.000	1	0.002	28	0.053
phycisphaera	58	0.103	1	0.002	5	0.008	122	0.232
clostridium	19	0.034	73	0.119	20	0.033	2	0.004
congregibacter	19	0.034	0	0.000	34	0.057	14	0.027
thauera	725	1.286	87	0.142	587	0.978	764	1.450
actinopolyspora	0	0.000	16	0.026	5	0.008	0	0.000
pseudohongiella	15	0.027	0	0.000	2	0.003	0	0.000
luedemannella	24	0.043	10	0.016	45	0.075	13	0.025
frankia	292	0.518	51	0.083	6	0.010	16	0.030
noviherbaspirillum	41	0.073	68	0.111	92	0.153	116	0.220
myxococcus	39	0.069	21	0.034	24	0.040	26	0.049
syntrophothermus	2	0.004	0	0.000	2	0.003	25	0.047
nevskia	15	0.027	0	0.000	0	0.000	43	0.082
arenimonas	27	0.048	0	0.000	92	0.153	10	0.019
methylobacterium	141	0.250	198	0.324	193	0.322	234	0.444
acidipila	0	0.000	46	0.075	39	0.065	0	0.000
azohydromonas	61	0.108	35	0.057	52	0.087	66	0.125

Table S6. Bacterial genera count and relative abundance across sampling sites

Genus	AXIS 1-2K		AXIS 1-4K		AXIS 2-4K		Remediation Site	
	Count	Relative Abundance	Count	Relative Abundance	Count	Relative Abundance	Count	Relative Abundance
ornatilinea	468	0.830	45	0.074	150	0.250	179	0.340
rhodococcus	164	0.291	1	0.002	18	0.030	5	0.009
terrimonas	584	1.036	35	0.057	110	0.183	550	1.044
pelobacter	203	0.360	6	0.010	71	0.118	230	0.437
hoeflea	4	0.007	0	0.000	32	0.053	3	0.006
sandaracinus	39	0.069	9	0.015	52	0.087	27	0.051
ramlibacter	12	0.021	2	0.003	37	0.062	41	0.078
blastococcus	140	0.248	404	0.661	330	0.550	713	1.353
tetrasphaera	28	0.050	65	0.106	170	0.283	10	0.019
fictibacillus	26	0.046	8	0.013	2	0.003	0	0.000
renibacterium	35	0.062	52	0.085	368	0.613	31	0.059
crinalium	0	0.000	36	0.059	0	0.000	0	0.000
tepidamorphus	1	0.002	19	0.031	7	0.012	2	0.004
rubellimicrobium	18	0.032	3	0.005	17	0.028	1	0.002
modestobacter	66	0.117	130	0.213	129	0.215	305	0.579
roseimicrobium	20	0.035	0	0.000	3	0.005	23	0.044
hydrogenophaga	16	0.028	22	0.036	50	0.083	34	0.065
thermomonas	21	0.037	3	0.005	130	0.217	5	0.009
intrasporangium	531	0.942	96	0.157	377	0.628	15	0.028
asticcacaulis	3	0.005	0	0.000	39	0.065	0	0.000
aridibacter	6315	11.202	577	0.944	926	1.543	2320	4.403
rhodopseudomonas	3	0.005	0	0.000	28	0.047	2	0.004
elstera	16	0.028	0	0.000	2	0.003	45	0.085
thermogemmatispora	0	0.000	1147	1.877	182	0.303	94	0.178
methylococcus	20	0.035	0	0.000	2	0.003	10	0.019
flavobacterium	40	0.071	2	0.003	4	0.007	10	0.019
aquisphaera	0	0.000	279	0.457	83	0.138	2	0.004
effusibacillus	24	0.043	0	0.000	6	0.010	7	0.013
pedobacter	0	0.000	0	0.000	18	0.030	0	0.000
ralstonia	0	0.000	27	0.044	42	0.070	3	0.006
bauldia	9	0.016	43	0.070	41	0.068	76	0.144
limimonas	122	0.216	109	0.178	52	0.087	146	0.277
thiocystis	4	0.007	0	0.000	15	0.025	15	0.028
janthinobacterium	0	0.000	0	0.000	23	0.038	0	0.000
oxalicibacterium	2	0.004	17	0.028	10	0.017	18	0.034
chelatococcus	9	0.016	8	0.013	49	0.082	142	0.270
candidatus koribacter	40	0.071	2750	4.500	1970	3.283	762	1.446
amycolatopsis	43	0.076	52	0.085	39	0.065	133	0.252
knoellia	89	0.158	96	0.157	402	0.670	40	0.076
thioalkalivibrio	10	0.018	0	0.000	0	0.000	6	0.011
hirschia	43	0.076	0	0.000	21	0.035	28	0.053

Table S6. Bacterial genera count and relative abundance across sampling sites

Genus	AXIS 1-2K		AXIS 1-4K		AXIS 2-4K		Remediation Site	
	Count	Relative Abundance	Count	Relative Abundance	Count	Relative Abundance	Count	Relative Abundance
thermanaerothrix	302	0.536	138	0.226	248	0.413	212	0.402
ilumatobacter	17	0.030	1	0.002	6	0.010	9	0.017
geofilum	13	0.023	0	0.000	0	0.000	0	0.000
calditerricola	123	0.218	245	0.401	19	0.032	125	0.237
panacagrimonas	35	0.062	3	0.005	2	0.003	25	0.047
achromobacter	7	0.012	2	0.003	24	0.040	13	0.025
jhaorihella	4	0.007	1	0.002	13	0.022	0	0.000
acinetobacter	9	0.016	1	0.002	81	0.135	15	0.028
thermosporothrix	0	0.000	305	0.499	8	0.013	0	0.000
mitsuaria	26	0.046	5	0.008	23	0.038	42	0.080
bryocella	0	0.000	42	0.069	2	0.003	1	0.002
fimbriimonas	52	0.092	0	0.000	7	0.012	21	0.040
kribbella	37	0.066	96	0.157	264	0.440	144	0.273
desulfobulbus	3	0.005	47	0.077	87	0.145	114	0.216
stella	3	0.005	343	0.561	76	0.127	50	0.095
dyella	6	0.011	0	0.000	286	0.477	4	0.008
terrabacter	3	0.005	159	0.260	849	1.415	13	0.025
kocuria	60	0.106	7	0.011	24	0.040	4	0.008
catenulispora	33	0.059	46	0.075	67	0.112	8	0.015
kouleothrix	237	0.420	147	0.241	160	0.267	199	0.378
herbaspirillum	25	0.044	84	0.137	98	0.163	75	0.142
caldimonas	1	0.002	7	0.011	13	0.022	10	0.019
aetherobacter	41	0.073	44	0.072	59	0.098	45	0.085
azotobacter	14	0.025	0	0.000	7	0.012	0	0.000
desulfotalea	4	0.007	6	0.010	21	0.035	3	0.006
aggregicoccus	137	0.243	56	0.092	67	0.112	40	0.076
aeromicrobium	338	0.600	4	0.007	157	0.262	180	0.342
laribacter	0	0.000	0	0.000	15	0.025	12	0.023
anaplasma	10	0.018	16	0.026	40	0.067	32	0.061
reyranella	258	0.458	93	0.152	454	0.757	414	0.786
enhydrobacter	0	0.000	2	0.003	13	0.022	4	0.008
solirubrobacter	2216	3.931	1236	2.023	930	1.550	1742	3.306
flexibacter	630	1.117	2	0.003	25	0.042	87	0.165
duganella	7	0.012	3	0.005	3	0.005	9	0.017
desulfonatronum	79	0.140	3	0.005	39	0.065	33	0.063
hylemonella	23	0.041	11	0.018	38	0.063	23	0.044
virgisporangium	2	0.004	6	0.010	0	0.000	68	0.129
methylosinus	40	0.071	64	0.105	170	0.283	29	0.055
streptomyces	2566	4.552	699	1.144	2066	3.443	1531	2.906
mycobacterium	367	0.651	349	0.571	391	0.652	444	0.843
annamia	0	0.000	27	0.044	0	0.000	0	0.000

Table S6. Bacterial genera count and relative abundance across sampling sites

Genus	AXIS 1-2K		AXIS 1-4K		AXIS 2-4K		Remediation Site	
	Count	Relative Abundance	Count	Relative Abundance	Count	Relative Abundance	Count	Relative Abundance
pedosphaera	2364	4.193	844	1.381	785	1.308	1571	2.982
aquisalimonas	21	0.037	0	0.000	6	0.010	28	0.053
pelomonas	22	0.039	0	0.000	13	0.022	109	0.207
steroidobacter	696	1.235	19	0.031	239	0.398	477	0.905
patulibacter	0	0.000	28	0.046	23	0.038	0	0.000
albimonas	40	0.071	0	0.000	3	0.005	1	0.002
pullulanibacillus	0	0.000	149	0.244	53	0.088	0	0.000
leifsonia	3	0.005	4	0.007	16	0.027	140	0.266
propionibacterium	0	0.000	35	0.057	6	0.010	0	0.000
rudaea	0	0.000	29	0.047	47	0.078	3	0.006
flavihumibacter	107	0.190	0	0.000	16	0.027	4	0.008
ignavibacterium	17	0.030	0	0.000	7	0.012	16	0.030
rummeliibacillus	7	0.012	3	0.005	0	0.000	82	0.156
parasphingopyxis	21	0.037	0	0.000	7	0.012	8	0.015
rhodomicrobium	0	0.000	5	0.008	45	0.075	1	0.002
sphingopyxis	8	0.014	0	0.000	13	0.022	0	0.000
adhaeribacter	47	0.083	2	0.003	19	0.032	24	0.046
starria	7	0.012	0	0.000	73	0.122	0	0.000
enterococcus	28	0.050	0	0.000	8	0.013	12	0.023
candidatus nitrotoga	1	0.002	0	0.000	0	0.000	30	0.057
kyrpidia	0	0.000	13	0.021	40	0.067	0	0.000
levilinea	0	0.000	3	0.005	43	0.072	2	0.004
acidiphilium	0	0.000	32	0.052	21	0.035	0	0.000
dehalogenimonas	2	0.004	738	1.208	62	0.103	9	0.017
jeotgalibacillus	2	0.004	1	0.002	6	0.010	4	0.008
thermobispora	83	0.147	4	0.007	15	0.025	44	0.084
telmatobacter	0	0.000	122	0.200	115	0.192	46	0.087
kallotenue	0	0.000	0	0.000	0	0.000	21	0.040
bythopirellula	53	0.094	0	0.000	18	0.030	110	0.209
deferrisoma	0	0.000	83	0.136	30	0.050	5	0.009
sphingobium	32	0.057	0	0.000	1	0.002	2	0.004
erythromicrobium	10	0.018	0	0.000	4	0.007	4	0.008
candidatus alysiosphaera	16	0.028	1	0.002	16	0.027	1	0.002
mechercharimyces	27	0.048	12	0.020	56	0.093	29	0.055
methylogaea	283	0.502	6	0.010	83	0.138	104	0.197
xanthobacter	14	0.025	13	0.021	15	0.025	24	0.046
pseudolabrys	321	0.569	309	0.506	854	1.423	830	1.575
woodsholea	157	0.278	1	0.002	16	0.027	91	0.173
sporosarcina	13	0.023	195	0.319	38	0.063	0	0.000
lapillicoccus	25	0.044	19	0.031	159	0.265	1	0.002
rhodothalassium	26	0.046	0	0.000	0	0.000	0	0.000

Table S6. Bacterial genera count and relative abundance across sampling sites

Genus	AXIS 1-2K		AXIS 1-4K		AXIS 2-4K		Remediation Site	
	Count	Relative Abundance	Count	Relative Abundance	Count	Relative Abundance	Count	Relative Abundance
parasegetibacter	120	0.213	4	0.007	31	0.052	122	0.232
rhizomicrobium	106	0.188	53	0.087	288	0.480	335	0.636
flavisolibacter	439	0.779	100	0.164	150	0.250	279	0.530
cystobacter	748	1.327	84	0.137	96	0.160	142	0.270
jatrophihabitans	1	0.002	196	0.321	118	0.197	19	0.036
chthoniobacter	76	0.135	478	0.782	345	0.575	942	1.788
geobacter	94	0.167	23	0.038	239	0.398	84	0.159
pantoea	8	0.014	9	0.015	15	0.025	28	0.053
chryseolinea	706	1.252	0	0.000	40	0.067	377	0.716
fodinibacter	56	0.099	0	0.000	3	0.005	0	0.000
bellilinea	281	0.498	1	0.002	13	0.022	4	0.008
thermoanaerobaculum	65	0.115	2	0.003	16	0.027	30	0.057
nitrosovibrio	975	1.729	167	0.273	954	1.590	1462	2.775
sphaerobacter	325	0.576	225	0.368	528	0.880	201	0.381
zavarzinella	98	0.174	222	0.363	92	0.153	243	0.461
acidicaldus	0	0.000	15	0.025	21	0.035	0	0.000
skermanella	507	0.899	108	0.177	164	0.273	689	1.308
aciditerrimonas	426	0.756	546	0.893	384	0.640	623	1.182
exiguobacterium	0	0.000	1	0.002	44	0.073	0	0.000
luteibacter	72	0.128	3	0.005	4	0.007	82	0.156
xylophilus	84	0.149	51	0.083	131	0.218	160	0.304
ammoniphilus	6	0.011	553	0.905	242	0.403	41	0.078
thermovum	1	0.002	1	0.002	0	0.000	22	0.042
pseudoxanthomonas	52	0.092	45	0.074	70	0.117	6	0.011
saccharopolyspora	1274	2.260	221	0.362	31	0.052	519	0.985
nocardia	0	0.000	7	0.011	16	0.027	56	0.106
dokdonella	7	0.012	8	0.013	32	0.053	22	0.042
thermaerobacter	9	0.016	9	0.015	1	0.002	49	0.093
chitinimonas	115	0.204	74	0.121	76	0.127	41	0.078
alsobacter	2	0.004	51	0.083	37	0.062	1	0.002
bacillus	1622	2.877	12007	19.648	2891	4.818	541	1.027
alicyclobacillus	36	0.064	394	0.645	220	0.367	0	0.000
gulbenkiania	31	0.055	0	0.000	0	0.000	0	0.000
kibdelosporangium	0	0.000	14	0.023	3	0.005	8	0.015
enhygromyxa	26	0.046	0	0.000	14	0.023	3	0.006
listeria	0	0.000	44	0.072	31	0.052	1	0.002
bryobacter	138	0.245	93	0.152	87	0.145	116	0.220
leptolinea	61	0.108	1	0.002	5	0.008	43	0.082
lacibacterium	20	0.035	0	0.000	68	0.113	34	0.065
nocardioides	1063	1.886	371	0.607	1398	2.330	394	0.748
aneurinibacillus	0	0.000	0	0.000	1	0.002	66	0.125

Table S6. Bacterial genera count and relative abundance across sampling sites

Genus	AXIS 1-2K		AXIS 1-4K		AXIS 2-4K		Remediation Site	
	Count	Relative Abundance	Count	Relative Abundance	Count	Relative Abundance	Count	Relative Abundance
rhodopila	2	0.004	409	0.669	138	0.230	35	0.066
sinomonas	21	0.037	177	0.290	46	0.077	37	0.070
acidovorax	134	0.238	87	0.142	240	0.400	247	0.469
thiobacter	47	0.083	127	0.208	322	0.537	288	0.547
tumebacillus	0	0.000	381	0.623	274	0.457	1	0.002
algiphilus	264	0.468	0	0.000	6	0.010	7	0.013
mesorhizobium	75	0.133	54	0.088	363	0.605	168	0.319
ardenticatena	0	0.000	0	0.000	0	0.000	68	0.129
lysinimonas	41	0.073	0	0.000	56	0.093	40	0.076
rhizobium	18	0.032	26	0.043	176	0.293	80	0.152
desulfacinum	58	0.103	15	0.025	143	0.238	107	0.203
allobacillus	1	0.002	3	0.005	23	0.038	5	0.009
edaphobacter	26	0.046	50	0.082	136	0.227	74	0.140
methylocystis	6	0.011	4	0.007	88	0.147	3	0.006
hydrogenibacillus	0	0.000	69	0.113	7	0.012	2	0.004
methyloceanibacter	12	0.021	3	0.005	0	0.000	0	0.000
conexibacter	530	0.940	3244	5.308	841	1.402	1555	2.951
alterococcus	22	0.039	0	0.000	0	0.000	11	0.021
caldilinea	52	0.092	59	0.097	19	0.032	5	0.009
piscinibacter	78	0.138	43	0.070	118	0.197	148	0.281
ancylobacter	15	0.027	5	0.008	18	0.030	45	0.085
hyphomonas	32	0.057	0	0.000	21	0.035	26	0.049
methyloversatilis	73	0.129	1	0.002	0	0.000	65	0.123
acidibacter	341	0.605	198	0.324	347	0.578	667	1.266
kineosporia	123	0.218	137	0.224	34	0.057	124	0.235
angustibacter	0	0.000	38	0.062	97	0.162	416	0.790
desulfuromonas	3	0.005	0	0.000	15	0.025	48	0.091
thermosinus	0	0.000	316	0.517	22	0.037	0	0.000
nitrosospira	28	0.050	50	0.082	210	0.350	128	0.243
agrobacterium	18	0.032	12	0.020	75	0.125	29	0.055
solitalea	53	0.094	2	0.003	18	0.030	217	0.412
actinophytocola	14	0.025	12	0.020	8	0.013	38	0.072
rubritepida	0	0.000	27	0.044	10	0.017	4	0.008
beijerinckia	2	0.004	66	0.108	16	0.027	8	0.015
iamia	272	0.482	5	0.008	25	0.042	93	0.177
sorangium	42	0.074	25	0.041	34	0.057	15	0.028
fulvimonas	17	0.030	0	0.000	7	0.012	4	0.008
phenylobacterium	199	0.353	118	0.193	115	0.192	89	0.169
curvibacter	0	0.000	0	0.000	59	0.098	0	0.000
smaragdicoccus	2	0.004	7	0.011	19	0.032	7	0.013
azonexus	125	0.222	0	0.000	5	0.008	36	0.068

Table S6. Bacterial genera count and relative abundance across sampling sites

Genus	AXIS 1-2K		AXIS 1-4K		AXIS 2-4K		Remediation Site	
	Count	Relative Abundance	Count	Relative Abundance	Count	Relative Abundance	Count	Relative Abundance
nitrospirillum	385	0.683	250	0.409	176	0.293	292	0.554
neorhizobium	1	0.002	0	0.000	17	0.028	1	0.002
empedobacter	0	0.000	45	0.074	7	0.012	1	0.002
chthonomonas	1	0.002	31	0.051	15	0.025	11	0.021
sediminibacterium	7	0.012	63	0.103	10	0.017	145	0.275
sporichthya	47	0.083	13	0.021	21	0.035	30	0.057
kofleria	58	0.103	45	0.074	44	0.073	75	0.142
amorphus	0	0.000	0	0.000	21	0.035	23	0.044
phaselicystis	20	0.035	0	0.000	1	0.002	9	0.017
gemmata	248	0.440	315	0.515	197	0.328	534	1.013
rhodocyclus	6	0.011	231	0.378	114	0.190	1	0.002
Total count	56376		61111		60002		52690	

Axis 1-2K: residential site, Axis 2-4K: agricultural dry land, Axis 2-4K: agricultural wet land, and REM: Remediated tailing

Table S7. Fungal genera count and relative abundance across sampling sites.

Genus	AXIS1-2K		AXIS 1-4K		AXIS 2-4K		Remediation Site	
	Count	Relative Abundance	Count	Relative Abundance	Count	Relative Abundance	Count	Relative Abundance
crepidotus	1205	0.631	24	0.014	4	0.011	26	0.016
mycena	136	0.071	39	0.023	357	0.979	479	0.299
oligoporus	0	0.000	0	0.000	16	0.044	0	0.000
gloiocephala	436	0.228	20	0.012	14	0.038	13	0.008
cosmospora	341	0.179	1732	1.030	65	0.178	95	0.059
ampulloclitocybe	13	0.007	5	0.003	0	0.000	88	0.055
hydropisphaera	116	0.061	14	0.008	24	0.066	26	0.016
rhizocarpon	19	0.010	447	0.266	5	0.014	49	0.031
leucogyrophana	39	0.020	5	0.003	20	0.055	54	0.034
eutypella	14	0.007	8	0.005	0	0.000	153	0.096
phlyctochytrium	30	0.016	52	0.031	3	0.008	214	0.134
lecanosticta	35	0.018	807	0.480	14	0.038	805	0.503
piloderma	83	0.043	1	0.001	0	0.000	4	0.002
leucoagaricus	37	0.019	3	0.002	0	0.000	2	0.001
thanatephorus	135	0.071	3636	2.163	14	0.038	257	0.160
rhizophagus	1984	1.039	1502	0.893	750	2.058	2357	1.472
pleiochaeta	3	0.002	3	0.002	0	0.000	20	0.012
articulospora	33	0.017	115	0.068	9	0.025	23	0.014
acremonium	1477	0.774	867	0.516	232	0.636	3276	2.046
paurocotylis	69	0.036	2	0.001	0	0.000	3	0.002
paraglomus	117	0.061	148	0.088	14	0.038	964	0.602
terfezia	1943	1.018	220	0.131	15	0.041	79	0.049
lodderomyces	356	0.187	10	0.006	4	0.011	14	0.009
lecanicillium	24	0.013	6	0.004	5	0.014	84	0.052
boeremia	4	0.002	33	0.020	0	0.000	1	0.001
microbotryum	102	0.053	7	0.004	1	0.003	11	0.007
rickenella	3	0.002	52	0.031	2	0.005	2	0.001
waitea	6	0.003	18	0.011	66	0.181	17	0.011
bagnisiella	2	0.001	2	0.001	0	0.000	18	0.011
hygrophorus	6	0.003	3	0.002	2	0.005	94	0.059
paraconiothyrium	212	0.111	491	0.292	87	0.239	2111	1.318
veronaea	7	0.004	7	0.004	24	0.066	117	0.073
hypholoma	5	0.003	51	0.030	0	0.000	1	0.001
elsinoe	1	0.001	0	0.000	0	0.000	16	0.010
epicoccum	314	0.165	2332	1.387	644	1.767	381	0.238
hymenochaete	3	0.002	3	0.002	18	0.049	41	0.026
holtermannia	106	0.056	5	0.003	3	0.008	1	0.001

Table S7. Fungal genera count and relative abundance across sampling sites.

Genus	AXIS1-2K		AXIS 1-4K		AXIS 2-4K		Remediation Site	
	Count	Relative Abundance	Count	Relative Abundance	Count	Relative Abundance	Count	Relative Abundance
cylindrocarpon	35	0.018	48	0.029	294	0.807	18	0.011
subplenodomus	4	0.002	4	0.002	1	0.003	180	0.112
massaria	852	0.446	480	0.285	27	0.074	85	0.053
entoloma	245	0.128	6	0.004	0	0.000	14	0.009
phaeosphaeriopsis	97	0.051	143	0.085	16	0.044	395	0.247
micarea	13	0.007	0	0.000	0	0.000	7	0.004
cladorrhinum	713	0.374	1236	0.735	444	1.218	1783	1.113
lepiota	10	0.005	106	0.063	199	0.546	8	0.005
magnaporthe	7	0.004	54	0.032	0	0.000	1	0.001
retroconis	49	0.026	10	0.006	12	0.033	2	0.001
candida	58	0.030	177	0.105	2	0.005	17	0.011
pseudoidriella	19	0.010	2	0.001	4	0.011	3	0.002
exidia	4	0.002	193	0.115	2	0.005	8	0.005
pseudorhizidium	1	0.001	4	0.002	41	0.112	7	0.004
sarea	125	0.065	93	0.055	15	0.041	2667	1.665
mycosphaerella	2	0.001	76	0.045	0	0.000	41	0.026
calcarisporiella	1	0.001	21	0.012	0	0.000	9	0.006
zopfiella	616	0.323	70	0.042	9	0.025	358	0.224
hyphodermella	0	0.000	9	0.005	0	0.000	0	0.000
lobulomyces	51	0.027	285	0.170	103	0.283	35	0.022
kodamaea	3	0.002	196	0.117	0	0.000	7	0.004
verpa	0	0.000	15	0.009	0	0.000	0	0.000
piriformospora	40	0.021	134	0.080	33	0.091	6	0.004
metacordyceps	293	0.153	63	0.037	154	0.422	258	0.161
leptospora	9	0.005	4	0.002	0	0.000	39	0.024
medicopsis	1	0.001	0	0.000	0	0.000	17	0.011
cookeina	445	0.233	212	0.126	4	0.011	40	0.025
chaetomium	307	0.161	1484	0.883	251	0.689	669	0.418
phlebopus	1	0.001	44	0.026	0	0.000	2	0.001
edenia	50	0.026	23	0.014	2	0.005	29	0.018
helicoma	423	0.222	4560	2.712	789	2.165	262	0.164
helminthosporium	5	0.003	126	0.075	8	0.022	6	0.004
sebacina	36	0.019	10	0.006	2	0.005	133	0.083
phlogicylindrium	0	0.000	17	0.010	0	0.000	3	0.002
spongipellis	3	0.002	121	0.072	0	0.000	6	0.004
dothidea	1	0.001	65	0.039	0	0.000	5	0.003
sistotrema	0	0.000	101	0.060	0	0.000	5	0.003
wickerhamia	13	0.007	3	0.002	0	0.000	0	0.000
Monochaetia	2	0.001	0	0.000	37	0.102	1	0.001
tubeufia	38	0.020	3	0.002	4	0.011	3	0.002

Table S7. Fungal genera count and relative abundance across sampling sites.

Genus	AXIS1-2K		AXIS 1-4K		AXIS 2-4K		Remediation Site	
	Count	Relative Abundance	Count	Relative Abundance	Count	Relative Abundance	Count	Relative Abundance
hysterium	34	0.018	1	0.001	8	0.022	10	0.006
heterobasidion	3	0.002	51	0.030	1	0.003	3	0.002
stromatonectria	32	0.017	19	0.011	1256	3.446	32	0.020
rhizophydium	17	0.009	611	0.363	0	0.000	18	0.011
athelia	41	0.021	2	0.001	0	0.000	0	0.000
rhizopus	17	0.009	197	0.117	0	0.000	13	0.008
gymnascella	69	0.036	12	0.007	0	0.000	4	0.002
archaeorhizomyces	1	0.001	1	0.001	0	0.000	19	0.012
derxomyces	3	0.002	53	0.032	8	0.022	0	0.000
stephanonectria	1	0.001	0	0.000	81	0.222	1	0.001
fimetariella	29	0.015	40	0.024	6	0.016	367	0.229
pseudocosmospora	454	0.238	90	0.054	14	0.038	33	0.021
isaria	23	0.012	2	0.001	0	0.000	73	0.046
sordaria	2447	1.282	1013	0.603	117	0.321	153	0.096
sarcoleotia	1	0.001	1	0.001	0	0.000	23	0.014
minimedusa	80	0.042	10	0.006	2	0.005	0	0.000
elmerina	489	0.256	25	0.015	16	0.044	156	0.097
coniella	0	0.000	1	0.001	31	0.085	6	0.004
perenniporia	2	0.001	26	0.015	53	0.145	5	0.003
ascotaiwania	390	0.204	18	0.011	3	0.008	39	0.024
aporospora	29	0.015	50	0.030	12	0.033	400	0.250
blastobotrys	31	0.016	1	0.001	0	0.000	48	0.030
rhodoveronaea	0	0.000	9	0.005	0	0.000	4	0.002
myceliophthora	37	0.019	3	0.002	0	0.000	2	0.001
bipolaris	107	0.056	1683	1.001	68	0.187	200	0.125
scytalidium	199	0.104	43	0.026	7	0.019	163	0.102
hypomyces	30	0.016	347	0.206	44	0.121	54	0.034
aureobasidium	13	0.007	83	0.049	2	0.005	32	0.020
fusidium	91	0.048	33	0.020	47	0.129	336	0.210
allomyces	119	0.062	87	0.052	3	0.008	16	0.010
rhizoctonia	2429	1.273	800	0.476	116	0.318	2555	1.595
gliomastix	201	0.105	13	0.008	35	0.096	7	0.004
cylindrocladiella	16	0.008	13	0.008	329	0.903	14	0.009
pyricularia	45	0.024	1	0.001	0	0.000	2	0.001
gymnostellatospora	3	0.002	3	0.002	95	0.261	3	0.002
sphaerobolus	1	0.001	0	0.000	35	0.096	1	0.001
ascosphaera	81	0.042	17	0.010	3	0.008	8	0.005
powellomyces	0	0.000	28	0.017	0	0.000	1	0.001

Table S7. Fungal genera count and relative abundance across sampling sites.

Genus	AXIS1-2K		AXIS 1-4K		AXIS 2-4K		Remediation Site	
	Count	Relative Abundance	Count	Relative Abundance	Count	Relative Abundance	Count	Relative Abundance
auxarthron	227	0.119	74	0.044	8	0.022	1649	1.030
hebeloma	1	0.001	1	0.001	0	0.000	12	0.007
schizosaccharomyces	10	0.005	226	0.134	1	0.003	5	0.003
curreya	1	0.001	2	0.001	0	0.000	24	0.015
stagonosporopsis	64	0.034	99	0.059	95	0.261	272	0.170
wettsteinina	11	0.006	356	0.212	1	0.003	14	0.009
diversispora	1220	0.639	135	0.080	27	0.074	514	0.321
periconia	30	0.016	243	0.145	7	0.019	323	0.202
anthostomella	13	0.007	179	0.106	1	0.003	19	0.012
pseudorobillarda	8	0.004	3	0.002	0	0.000	117	0.073
ochroconis	27	0.014	106	0.063	18	0.049	395	0.247
gymnopilus	27	0.014	38	0.023	5	0.014	6	0.004
veluticeps	9	0.005	0	0.000	1	0.003	1	0.001
phialocephala	100	0.052	327	0.194	14	0.038	19	0.012
chaetomella	3	0.002	1	0.001	32	0.088	1	0.001
conlarium	2	0.001	121	0.072	7	0.019	5	0.003
volvariella	1174	0.615	43	0.026	2	0.005	19	0.012
trichoderma	253	0.133	621	0.369	1471	4.036	125	0.078
acarospora	18	0.009	1	0.001	0	0.000	0	0.000
clitopilus	4	0.002	79	0.047	2	0.005	4	0.002
russula	117	0.061	5	0.003	22	0.060	3	0.002
achaetomium	6	0.003	2	0.001	0	0.000	44	0.027
leptosphaerulina	263	0.138	3469	2.063	224	0.615	1010	0.631
cephaliophora	2066	1.082	172	0.102	69	0.189	99	0.062
laetisaria	15	0.008	2	0.001	0	0.000	1	0.001
dacryopinax	83	0.043	1	0.001	8	0.022	2	0.001
calonectria	27	0.014	35	0.021	4	0.011	5	0.003
ramaria	26	0.014	2	0.001	39	0.107	0	0.000
coemansia	6	0.003	144	0.086	1	0.003	5	0.003
apodus	1700	0.891	117	0.070	40	0.110	68	0.042
neofusicoccum	1	0.001	0	0.000	0	0.000	30	0.019
phoma	7549	3.955	6322	3.760	1005	2.757	3341	2.086
cadophora	2	0.001	35	0.021	0	0.000	3	0.002
monocillium	1	0.001	4	0.002	4	0.011	32	0.020
torula	29	0.015	17	0.010	0	0.000	10	0.006
scleroderma	5	0.003	21	0.012	5	0.014	3	0.002
mucor	6	0.003	2	0.001	0	0.000	28	0.017
alternaria	398	0.209	417	0.248	75	0.206	4822	3.011
eucasphaeria	4	0.002	94	0.056	11	0.030	5	0.003

Table S7. Fungal genera count and relative abundance across sampling sites.

Genus	AXIS1-2K		AXIS 1-4K		AXIS 2-4K		Remediation Site	
	Count	Relative Abundance	Count	Relative Abundance	Count	Relative Abundance	Count	Relative Abundance
dinemasporium	8	0.004	2	0.001	1	0.003	75	0.047
lecidea	15	0.008	0	0.000	0	0.000	2	0.001
glomus	131	0.069	138	0.082	4	0.011	1245	0.777
phillipsia	17	0.009	267	0.159	3	0.008	10	0.006
toxicocladosporium	3	0.002	13	0.008	0	0.000	10	0.006
hyphopichia	71	0.037	57	0.034	33	0.091	14	0.009
ossicaulis	14	0.007	146	0.087	0	0.000	33	0.021
protrudomyces	172	0.090	9	0.005	1	0.003	33	0.021
clavispora	3	0.002	54	0.032	0	0.000	1	0.001
saccharata	16	0.008	0	0.000	0	0.000	0	0.000
psathyrella	148	0.078	12	0.007	148	0.406	7	0.004
tricharina	16	0.008	4	0.002	6	0.016	19	0.012
hannaella	94	0.049	319	0.190	11	0.030	672	0.420
paraphaeosphaeria	24	0.013	82	0.049	77	0.211	11	0.007
passalora	2	0.001	21	0.012	0	0.000	2	0.001
mariannaea	7	0.004	88	0.052	7	0.019	31	0.019
phaeoisaria	6	0.003	0	0.000	2	0.005	14	0.009
trichothecium	256	0.134	46	0.027	6	0.016	546	0.341
lichtheimia	14	0.007	396	0.236	1	0.003	22	0.014
pleurothecium	5	0.003	9	0.005	1	0.003	119	0.074
corynespora	145	0.076	24	0.014	2	0.005	11	0.007
tranzschelia	9	0.005	13	0.008	1	0.003	1	0.001
cephalosporium	0	0.000	18	0.011	0	0.000	18	0.011
panaeolus	952	0.499	23	0.014	2	0.005	26	0.016
rigidoporus	28	0.015	1	0.001	1	0.003	68	0.042
leptodiscella	28	0.015	30	0.018	25	0.069	327	0.204
leptosphaeria	0	0.000	5	0.003	5	0.014	18	0.011
galerina	8	0.004	6	0.004	0	0.000	102	0.064
arthrobotrys	443	0.232	128	0.076	95	0.261	85	0.053
cyphellophora	66	0.035	19	0.011	27	0.074	413	0.258
ilyonectria	24	0.013	1	0.001	49	0.134	5	0.003
serendipita	571	0.299	33	0.020	19	0.052	582	0.363
bambusicola	37	0.019	174	0.103	11	0.030	314	0.196
rhabdocline	24	0.013	0	0.000	0	0.000	0	0.000
dipodascus	32	0.017	1039	0.618	13	0.036	36	0.022
engyodontium	893	0.468	104	0.062	29	0.080	94	0.059
aspergillus	7447	3.901	1744	1.037	334	0.916	4146	2.589
myrothecium	671	0.352	259	0.154	248	0.680	1071	0.669
pulvinula	5	0.003	10	0.006	0	0.000	91	0.057
zancudomyces	33	0.017	111	0.066	5	0.014	714	0.446

Table S7. Fungal genera count and relative abundance across sampling sites.

Genus	AXIS1-2K		AXIS 1-4K		AXIS 2-4K		Remediation Site	
	Count	Relative Abundance	Count	Relative Abundance	Count	Relative Abundance	Count	Relative Abundance
marssonina	4	0.002	32	0.019	0	0.000	2	0.001
dominikia	160	0.084	86	0.051	2	0.005	345	0.215
emericella	16	0.008	167	0.099	4	0.011	20	0.012
pulchromyces	1	0.001	52	0.031	0	0.000	4	0.002
acaulospora	125	0.065	65	0.039	4	0.011	6	0.004
shiraia	46	0.024	4	0.002	1	0.003	19	0.012
termitomyces	8	0.004	7	0.004	7	0.019	8	0.005
falcocladium	5	0.003	11	0.007	1	0.003	80	0.050
heterochaete	984	0.516	95	0.057	10	0.027	54	0.034
kockovaella	2	0.001	0	0.000	0	0.000	14	0.009
meliniomyces	10	0.005	280	0.167	1	0.003	48	0.030
allantophomopsis	0	0.000	68	0.040	1	0.003	2	0.001
graphium	64	0.034	7	0.004	1	0.003	1	0.001
diplodia	1	0.001	37	0.022	1	0.003	6	0.004
tarzetta	206	0.108	29	0.017	5	0.014	431	0.269
chloridium	11	0.006	97	0.058	68	0.187	12	0.007
cystobasidium	2	0.001	55	0.033	0	0.000	3	0.002
metarhizium	94	0.049	6	0.004	0	0.000	3	0.002
spirosphaera	417	0.218	16	0.010	0	0.000	15	0.009
phanerochaete	1	0.001	38	0.023	0	0.000	3	0.002
exophiala	525	0.275	199	0.118	544	1.492	2269	1.417
chaetomidium	469	0.246	38	0.023	26	0.071	75	0.047
trichocladium	51	0.027	12	0.007	4	0.011	82	0.051
aphanoascus	2	0.001	2	0.001	38	0.104	0	0.000
plectosphaerella	1572	0.824	867	0.516	1818	4.988	1620	1.012
gaeumannomyces	109	0.057	73	0.043	0	0.000	36	0.022
monosporascus	60	0.031	4	0.002	1	0.003	42	0.026
saccharicola	649	0.340	115	0.068	8	0.022	38	0.024
botryosphaeria	23	0.012	468	0.278	3	0.008	36	0.022
cladosporium	1043	0.546	2216	1.318	857	2.351	7519	4.695
verticillium	41	0.021	6	0.004	1	0.003	3	0.002
septoria	164	0.086	923	0.549	60	0.165	121	0.076
sporormiella	18	0.009	4	0.002	18	0.049	179	0.112
ramularia	19	0.010	16	0.010	13	0.036	137	0.086
leucosphaerina	8	0.004	1	0.001	2	0.005	40	0.025
notholepiota	4	0.002	177	0.105	6	0.016	2	0.001
peziza	76	0.040	42	0.025	1	0.003	1	0.001
thielavia	286	0.150	1157	0.688	146	0.401	92	0.057
coniosporium	3	0.002	28	0.017	2	0.005	32	0.020
podospora	2038	1.068	189	0.112	86	0.236	140	0.087
paradictyoarthrinium	2	0.001	2	0.001	1	0.003	13	0.008

Table S7. Fungal genera count and relative abundance across sampling sites.

Genus	AXIS1-2K		AXIS 1-4K		AXIS 2-4K		Remediation Site	
	Count	Relative Abundance	Count	Relative Abundance	Count	Relative Abundance	Count	Relative Abundance
staphylotrichum	4	0.002	18	0.011	0	0.000	1	0.001
amanita	2	0.001	41	0.024	0	0.000	2	0.001
mortierella	25741	13.485	1153	0.686	855	2.346	1175	0.734
camarographium	61	0.032	77	0.046	4	0.011	944	0.589
arthrinium	7	0.004	56	0.033	139	0.381	16	0.010
coniochaeta	18	0.009	6	0.004	0	0.000	0	0.000
pseudofavolus	4	0.002	4	0.002	2	0.005	49	0.031
rhizophlyctis	387	0.203	4019	2.390	212	0.582	2078	1.298
scolecobasidium	29	0.015	559	0.332	307	0.842	104	0.065
chrysosporium	566	0.297	131	0.078	515	1.413	34	0.021
malbranchea	11	0.006	46	0.027	7	0.019	2	0.001
peyronellaea	218	0.114	622	0.370	262	0.719	189	0.118
myrmecridium	332	0.174	31	0.018	49	0.134	55	0.034
peniophorella	5	0.003	4	0.002	3	0.008	192	0.120
radulidium	1	0.001	21	0.012	18	0.049	10	0.006
clitocella	0	0.000	0	0.000	34	0.093	0	0.000
adisciso	1	0.001	37	0.022	0	0.000	4	0.002
phaeophleospora	28	0.015	230	0.137	3	0.008	382	0.239
claroideoglomus	38	0.020	223	0.133	2	0.005	65	0.041
magnaporthiopsis	14	0.007	183	0.109	2	0.005	11	0.007
spizellomyces	2649	1.388	7994	4.755	202	0.554	1053	0.658
vermispora	5	0.003	6	0.004	0	0.000	112	0.070
didymosphaeria	3	0.002	8	0.005	2	0.005	12	0.007
schizopora	1	0.001	0	0.000	27	0.074	1	0.001
nectria	134	0.070	48	0.029	72	0.198	213	0.133
conocybe	4704	2.464	102	0.061	79	0.217	84	0.052
dentiscutata	5	0.003	40	0.024	2	0.005	49	0.031
pestalotiopsis	20	0.010	50	0.030	226	0.620	14	0.009
clonostachys	486	0.255	2608	1.551	183	0.502	792	0.495
funneliformis	182	0.095	1301	0.774	14	0.038	718	0.448
lepidosphaeria	12	0.006	2	0.001	0	0.000	0	0.000
pseudogymnoascus	23	0.012	1	0.001	0	0.000	1	0.001
nephroma	56	0.029	268	0.159	5	0.014	112	0.070
pichia	31	0.016	0	0.000	2	0.005	1	0.001
coniophora	129	0.068	6	0.004	0	0.000	1	0.001
umbilicaria	37	0.019	70	0.042	5	0.014	970	0.606
setosphaeria	8	0.004	2	0.001	1	0.003	2	0.001
purpureocillium	26	0.014	28	0.017	7	0.019	384	0.240
phialophora	1657	0.868	98	0.058	145	0.398	52	0.032
didymella	4	0.002	15	0.009	3	0.008	1	0.001

Table S7. Fungal genera count and relative abundance across sampling sites.

Genus	AXIS1-2K		AXIS 1-4K		AXIS 2-4K		Remediation Site	
	Count	Relative Abundance	Count	Relative Abundance	Count	Relative Abundance	Count	Relative Abundance
gymnoascus	4	0.002	89	0.053	4	0.011	1	0.001
spiromastix	225	0.118	5068	3.014	95	0.261	210	0.131
schizothecium	46	0.024	655	0.390	18	0.049	40	0.025
neurospora	2	0.001	3	0.002	31	0.085	1	0.001
clydaea	170	0.089	700	0.416	60	0.165	225	0.141
danielozyma	7	0.004	254	0.151	0	0.000	7	0.004
pluteus	428	0.224	11	0.007	67	0.184	7	0.004
letendraea	5	0.003	22	0.013	11	0.030	5	0.003
phomopsis	1	0.001	1	0.001	0	0.000	14	0.009
inocybe	84	0.044	176	0.105	0	0.000	19	0.012
septoglomus	15	0.008	6	0.004	1	0.003	49	0.031
thyronectria	20	0.010	14	0.008	1	0.003	172	0.107
dendrophoma	30	0.016	152	0.090	58	0.159	191	0.119
choanephora	1	0.001	13	0.008	0	0.000	2	0.001
gaertneriomyces	2396	1.255	176	0.105	19	0.052	53	0.033
phellodon	2	0.001	2	0.001	158	0.433	3	0.002
lophotrichus	21	0.011	0	0.000	2	0.005	0	0.000
polyporus	5	0.003	5	0.003	1	0.003	375	0.234
racocetra	121	0.063	124	0.074	16	0.044	3821	2.386
lasiodiplodia	104	0.054	22	0.013	123	0.337	81	0.051
trechispora	28	0.015	14	0.008	88	0.241	140	0.087
sparassis	52	0.027	0	0.000	0	0.000	7	0.004
ramulispora	66	0.035	48	0.029	3	0.008	298	0.186
globomyces	9	0.005	166	0.099	115	0.315	11	0.007
stilbella	784	0.411	175	0.104	32	0.088	165	0.103
rhodosporidium	2	0.001	2	0.001	39	0.107	3	0.002
arthroderma	155	0.081	13	0.008	0	0.000	16	0.010
cladophialophora	138	0.072	1007	0.599	89	0.244	1958	1.223
microdochium	175	0.092	69	0.041	54	0.148	684	0.427
wallemia	19	0.010	99	0.059	9	0.025	60	0.037
colletotrichum	3122	1.636	92	0.055	15	0.041	259	0.162
vertexicola	6	0.003	99	0.059	1	0.003	4	0.002
marasmius	17	0.009	118	0.070	3	0.008	25	0.016
trichophyton	3	0.002	82	0.049	2	0.005	4	0.002
pseudeurotium	4	0.002	1	0.001	19	0.052	0	0.000
dactylella	0	0.000	14	0.008	0	0.000	1	0.001
sarocladium	335	0.176	154	0.092	37	0.102	2994	1.870
catenaria	1718	0.900	82	0.049	81	0.222	50	0.031
degelia	1	0.001	36	0.021	0	0.000	1	0.001
monacrosporium	27	0.014	0	0.000	0	0.000	1	0.001

Table S7. Fungal genera count and relative abundance across sampling sites.

Genus	AXIS1-2K		AXIS 1-4K		AXIS 2-4K		Remediation Site	
	Count	Relative Abundance	Count	Relative Abundance	Count	Relative Abundance	Count	Relative Abundance
smittium	6	0.003	0	0.000	0	0.000	98	0.061
tapinella	176	0.092	10	0.006	2	0.005	4	0.002
phaeosphaeria	2	0.001	4	0.002	0	0.000	34	0.021
amaurascopsis	4	0.002	226	0.134	1	0.003	13	0.008
genea	28	0.015	1	0.001	1	0.003	0	0.000
gelasinospora	92	0.048	68	0.040	9	0.025	285	0.178
cercophora	1010	0.529	88	0.052	46	0.126	538	0.336
ochrocladosporium	87	0.046	40	0.024	18	0.049	497	0.310
scedosporium	567	0.297	23	0.014	30	0.082	18	0.011
chaetosphaeria	0	0.000	16	0.010	46	0.126	4	0.002
ramicandelaber	5	0.003	13	0.008	13	0.036	96	0.060
monilinia	2	0.001	100	0.059	0	0.000	3	0.002
zymoseptoria	4	0.002	52	0.031	0	0.000	4	0.002
oliveonia	611	0.320	25	0.015	8	0.022	43	0.027
savoryella	55	0.029	5	0.003	0	0.000	1	0.001
lasiosphaeria	5	0.003	12	0.007	0	0.000	54	0.034
heydenia	160	0.084	7	0.004	14	0.038	3	0.002
bettsia	1	0.001	1	0.001	0	0.000	24	0.015
neoerysiphe	6	0.003	26	0.015	10	0.027	24	0.015
cinereomyces	761	0.399	20	0.012	1	0.003	13	0.008
coniothyrium	164	0.086	11	0.007	2	0.005	103	0.064
microascus	85	0.045	2	0.001	2	0.005	5	0.003
parascedosporium	12	0.006	1	0.001	26	0.071	0	0.000
dictyosporium	21	0.011	8	0.005	2	0.005	116	0.072
dactylaria	199	0.104	103	0.061	28	0.077	3335	2.083
lectera	253	0.133	2600	1.546	208	0.571	147	0.092
paecilomyces	10	0.005	9	0.005	1	0.003	69	0.043
ophiocordyceps	4262	2.233	258	0.153	81	0.222	2327	1.453
talaromyces	252	0.132	4674	2.780	256	0.702	224	0.140
gibellulopsis	414	0.217	49	0.029	166	0.455	112	0.070
beauveria	52	0.027	13	0.008	13	0.036	104	0.065
dokmaia	1790	0.938	1856	1.104	864	2.370	2568	1.604
lomentospora	21	0.011	3	0.002	6	0.016	0	0.000
geastrum	4	0.002	1	0.001	0	0.000	8	0.005
ustilaginoidea	3	0.002	3	0.002	2	0.005	16	0.010
massarina	11	0.006	13	0.008	276	0.757	4	0.002
pholiota	6	0.003	104	0.062	1	0.003	6	0.004
olpidium	55	0.029	23	0.014	16	0.044	2	0.001
agrocybe	33	0.017	6	0.004	3	0.008	41	0.026
macrophomina	430	0.225	1068	0.635	38	0.104	79	0.049

Table S7. Fungal genera count and relative abundance across sampling sites.

Genus	AXIS1-2K		AXIS 1-4K		AXIS 2-4K		Remediation Site	
	Count	Relative Abundance	Count	Relative Abundance	Count	Relative Abundance	Count	Relative Abundance
coprinopsis	229	0.120	12	0.007	20	0.055	14	0.009
campylocarpon	7	0.004	5	0.003	23	0.063	1	0.001
trichosporon	389	0.204	48	0.029	18	0.049	14	0.009
boothiomyces	0	0.000	19	0.011	0	0.000	1	0.001
xylaria	4	0.002	24	0.014	0	0.000	0	0.000
pseudoramichloridium	1	0.001	1	0.001	0	0.000	31	0.019
neosartorya	15	0.008	62	0.037	2	0.005	5	0.003
terramyces	1	0.001	16	0.010	0	0.000	1	0.001
nalanthamala	30	0.016	0	0.000	0	0.000	0	0.000
fusicolla	315	0.165	1916	1.140	99	0.272	464	0.290
cryptococcus	1424	0.746	5995	3.566	1236	3.391	2815	1.758
ganoderma	28	0.015	205	0.122	16	0.044	31	0.019
fusarium	38735	20.293	26300	15.642	7318	20.076	30005	18.737
lactarius	5	0.003	167	0.099	0	0.000	17	0.011
westerdykella	312	0.163	373	0.222	285	0.782	32	0.020
monographella	322	0.169	1055	0.627	36	0.099	75	0.047
curvularia	215	0.113	950	0.565	63	0.173	223	0.139
rhizopogon	15	0.008	17	0.010	0	0.000	2	0.001
tetraplosphaeria	3	0.002	10	0.006	49	0.134	21	0.013
acrocalymma	630	0.330	158	0.094	346	0.949	1887	1.178
diaporthe	266	0.139	32	0.019	4	0.011	187	0.117
alphamyces	62	0.032	41	0.024	6	0.016	35	0.022
myriodontium	63	0.033	28	0.017	0	0.000	497	0.310
kochiomyces	18	0.009	735	0.437	50	0.137	39	0.024
aporpium	0	0.000	43	0.026	0	0.000	2	0.001
taphrina	12	0.006	1	0.001	0	0.000	1	0.001
ascobolus	2788	1.461	101	0.060	54	0.148	67	0.042
stromatoneurospora	5	0.003	210	0.125	3	0.008	10	0.006
ascochyta	9	0.005	176	0.105	2	0.005	17	0.011
vascellum	19	0.010	14	0.008	24	0.066	37	0.023
pulcherricium	3	0.002	51	0.030	0	0.000	2	0.001
pycnoporellus	89	0.047	4	0.002	2	0.005	97	0.061
hamigera	46	0.024	4	0.002	0	0.000	5	0.003
leucocoprinus	24	0.013	12	0.007	43	0.118	400	0.250
hyalodendriella	0	0.000	25	0.015	0	0.000	2	0.001
tulostoma	61	0.032	19	0.011	1	0.003	6	0.004
xylogone	6	0.003	1	0.001	1	0.003	6	0.004
ophiostoma	48	0.025	20	0.012	1	0.003	3	0.002
pseudomassaria	1385	0.726	83	0.049	19	0.052	50	0.031
echria	121	0.063	542	0.322	14	0.038	37	0.023

Table S7. Fungal genera count and relative abundance across sampling sites.

Genus	AXIS1-2K		AXIS 1-4K		AXIS 2-4K		Remediation Site	
	Count	Relative Abundance	Count	Relative Abundance	Count	Relative Abundance	Count	Relative Abundance
humicola	9446	4.949	13712	8.155	1782	4.889	6974	4.355
boreostereum	1	0.001	0	0.000	46	0.126	0	0.000
lipomyces	21	0.011	193	0.115	2	0.005	115	0.072
cordyceps	445	0.233	213	0.127	29	0.080	452	0.282
parastagonospora	0	0.000	0	0.000	0	0.000	12	0.007
kurtzmanomyces	0	0.000	0	0.000	16	0.044	2	0.001
rhodotorula	3	0.002	4	0.002	194	0.532	5	0.003
phaeothecoidea	40	0.021	100	0.059	5	0.014	867	0.541
scleroramularia	12	0.006	213	0.127	107	0.294	9	0.006
sporobolomyces	6	0.003	18	0.011	4	0.011	8	0.005
munkovalsaria	1	0.001	8	0.005	0	0.000	9	0.006
coprinellus	2428	1.272	66	0.039	107	0.294	47	0.029
knufia	1921	1.006	5626	3.346	879	2.411	5702	3.561
chromosera	16	0.008	234	0.139	4	0.011	206	0.129
pleurotus	3	0.002	2	0.001	0	0.000	8	0.005
lentinula	326	0.171	13	0.008	8	0.022	12	0.007
corticium	1013	0.531	43	0.026	63	0.173	26	0.016
bullera	2	0.001	3	0.002	1	0.003	21	0.013
preussia	475	0.249	443	0.263	162	0.444	414	0.259
leiosphaerella	25	0.013	4	0.002	8	0.022	44	0.027
volutella	521	0.273	4202	2.499	161	0.442	272	0.170
metschnikowia	526	0.276	109	0.065	22	0.060	242	0.151
phialosimplex	5	0.003	94	0.056	3	0.008	6	0.004
pyrenochaeta	182	0.095	266	0.158	130	0.357	5016	3.132
tetracladium	9	0.005	7	0.004	1	0.003	79	0.049
hypocreopsis	9	0.005	10	0.006	0	0.000	2	0.001
ceratobasidium	40	0.021	47	0.028	2	0.005	398	0.249
corollospora	15	0.008	2	0.001	1	0.003	2	0.001
viridispora	10	0.005	89	0.053	6	0.016	12	0.007
dactylellina	113	0.059	30	0.018	1	0.003	231	0.144
scopulariopsis	69	0.036	51	0.030	1	0.003	294	0.184
phialemonium	7	0.004	70	0.042	44	0.121	73	0.046
acrostalagmus	36	0.019	3	0.002	0	0.000	2	0.001
amauroascus	2	0.001	1	0.001	0	0.000	48	0.030
dendryphion	2	0.001	0	0.000	0	0.000	27	0.017
penicillium	2346	1.229	3274	1.947	295	0.809	5767	3.601
nigrospora	220	0.115	159	0.095	27	0.074	357	0.223
robillarda	19	0.010	8	0.005	27	0.074	80	0.050
maunachytrium	264	0.138	218	0.130	8	0.022	19	0.012
austropaxillus	0	0.000	32	0.019	0	0.000	1	0.001

Table S7. Fungal genera count and relative abundance across sampling sites.

Genus	AXIS1-2K		AXIS 1-4K		AXIS 2-4K		Remediation Site	
	Count	Relative Abundance	Count	Relative Abundance	Count	Relative Abundance	Count	Relative Abundance
lycoperdon	237	0.124	9	0.005	70	0.192	29	0.018
lophiostoma	1505	0.788	257	0.153	128	0.351	258	0.161
agaricus	48	0.025	79	0.047	6	0.016	3	0.002
hygrocybe	223	0.117	246	0.146	25	0.069	15	0.009
drechslerella	30	0.016	1	0.001	0	0.000	0	0.000
stachybotrys	1769	0.927	390	0.232	89	0.244	139	0.087
phlebia	75	0.039	9	0.005	2	0.005	31	0.019
morchella	131	0.069	6	0.004	15	0.041	4	0.002
fomitiporia	0	0.000	35	0.021	3	0.008	1	0.001
microcera	41	0.021	310	0.184	11	0.030	26	0.016
strelitziana	31	0.016	85	0.051	4	0.011	338	0.211
stereum	6	0.003	375	0.223	16	0.044	7	0.004
tuber	38	0.020	1007	0.599	6	0.016	23	0.014
Total Count	190880		168132		36451		160140	

Axis 1-2K: residential site, Axis 2-4K: agricultural dry land, Axis 2-4K: agricultural wet land, and REM: Remediated tailing
